# Positively Charged Polymers as Promising Devices against Multidrug Resistant Gram-Negative Bacteria: A Review

**DOI:** 10.3390/polym12051195

**Published:** 2020-05-23

**Authors:** Silvana Alfei, Anna Maria Schito

**Affiliations:** 1 Department of Pharmacy (DiFAR), University of Genoa, Viale Cembrano 4, I-16148 Genova, Italy; 2 Department of Surgical Sciences and Integrated Diagnostics (DISC), University of Genoa, Viale Benedetto XV, 6, I-16132 Genova, Italy; amschito@unige.it

**Keywords:** antibiotic resistance, Gram-negative bacteria, hemolytic cytotoxicity, membrane disruption, positively charged polymers

## Abstract

Antibiotic resistance has increased markedly in Gram-negative bacteria, causing severe infections intractable with traditional drugs and amplifying mortality and healthcare costs. Consequently, to find novel antimicrobial compounds, active on multidrug resistant bacteria, is mandatory. In this regard, cationic antimicrobial peptides (CAMPs)—able to kill pathogens on contact—could represent an appealing solution. However, low selectivity, hemolytic toxicity and cost of manufacturing, hamper their massive clinical application. In the recent years—starting from CAMPs as template molecules—less toxic and lower-cost synthetic mimics of CAMPs, including cationic peptides, polymers and dendrimers, have been developed. Although the pending issue of hemolytic toxicity and biodegradability is still left not completely solved, cationic antimicrobial polymers (CAPs), compared to small drug molecules, thanks to their high molecular weight, own appreciable selectivity, reduced toxicity toward eukaryotic cells, more long-term activity, stability and non-volatility. With this background, an updated overview concerning the main manufactured types of CAPs, active on Gram-negative bacteria, is herein reported, including synthetic procedure and action’s mechanism. Information about their structures, antibacterial activity, advantages and drawbacks, was reported in the form of tables, which allow faster consultation and quicker learning concerning current CAPs state of the art, in order not to retrace reviews already available.

## 1. Introduction

The increasing replacement of antibiotic-susceptible bacteria (ASB) with antibiotic-resistant bacteria (ARB) is one of the most concern of microbiologists and over the last two decades, antibiotic resistance has increased markedly in Gram-negative bacteria and has determined an improvement of mortality and of healthcare costs [1,2].

Gram-negative bacteria pose a major threat to human health, since they are the most critically resistant and rapidly spreading bacteria, frequently responsible for severe and often deadly infections, not only in the general population, but also in the hospital settings or among people with weak or not yet fully developed immune systems, such as newborns, elderly, people undergoing surgery and cancer treatment.

Gram-negative bacteria, such as *Klebsiella pneumoniae, Acinetobacter baumanni, Pseudomonas aeruginosa, Burkholderia cepacia* and *Escherichia coli*, are responsible of severe infections including pneumonia, bloodstream infections, wound or surgical site infections and meningitis in healthcare settings [3].

Unfortunately, as very recently outlined by two reports published by the World Health Organization (WHO) on new antibiotic agents, among the 50 innovative molecules in development, very few target Gram-negative species [4,5].This findings raise deep concern, especially if considering a previous report published by WHO in 2017 [6] indicating 12 classes of bacteria that are highly critical for human health, due to their extraordinary resistant traits, where, in addition to *Mycobacterium tuberculosis*, Gram-negative pathogens clearly outnumber the Gram-positive ones. The prevalence of Gram-negative bacteria over Gram-positive is evident in all the priority groups identified in the report, such as the “other priority pathogens” group (where *A. baumannii, P. aeruginosa* and *Enterobacteriaceae* are included), the “high priority” group (encompassing *Helicobacter pylori*, Campylobacter specie, Salmonella species and *Neisseria gonorrhoeae*) and the “medium priority” group (that include *Hemophilus influenzae* and Shigella species).

Lastly, Gram-negative bacteria, unlike Gram-positive bacteria, are characterized by high and similar resistance levels, both in Europe and in the United States. In fact, citing the same report [6]: “when compared to the US data, the European Center for Disease Prevention and Control (ECDC) surveillance network showed overall lower rates of resistance in Gram-positive bacteria (although with large differences between countries) and the same worrying rates among Gram-negative bacteria”

These reports, developed by a WHO-led group of independent experts, encourage the medical research community to develop innovative treatments for these resistant Gram-negative bacteria, which are spreading rapidly and, more than Gram-positive ones, require urgent solutions.

Incessantly, Gram-negative bacteria build-in abilities, to find new ways to be resilient to drugs and are also able to pass along genetic materials that allow other bacteria to become drug-resistant as well [7]. Genotyping and sequencing the whole genome of large groups of isolated clinical bacterial has allowed the scientists to understand how antibiotic resistance develops and transmits both among bacteria and patients [8]. The most clinically important resistance phenotypes include carbapenem resistant *Enterobacteriaceae*, extensively drug resistant (XDR) *P. aeruginosa* and XRD *A. baumannii.*

New Delhi metallo-beta-lactamase 1 (NDM-1) makes bacteria resistant to a broad range of antibiotics, including those from the carbapenem family, which today are the last line of defense against antibiotic-resistant bacterial infections.

Antibiotic degradation, antibiotic target modification, modulation of permeability through the bacterial membrane and structural modifications of bacterial lipopolysaccharide are some of the established mechanisms of resistance and their knowledge have influenced the development of novel antibiotics for replacing ineffective beta lactams and have disposed innovative treatment practices in highly resistant infections [9].

It was established that the traditional antibiotics in the form of single target small molecules or small hydrophobic drugs, often fail in fighting multidrug resistant bacteria [10] and therefore the search for identifying structurally different and more effective forms of antimicrobial agents, active especially against Gram-negative strains is increasingly necessary and urgent.

In this regard, naturally occurring cationic antimicrobial peptides (CAMPs) are a wide well-performant class of not beta lactams antimicrobial agents [11,12,13], with a broad spectrum of action, active on a wide variety of Gram-positive and Gram-negative bacteria, fungi, protozoa and yeast.

**Figure 1:**
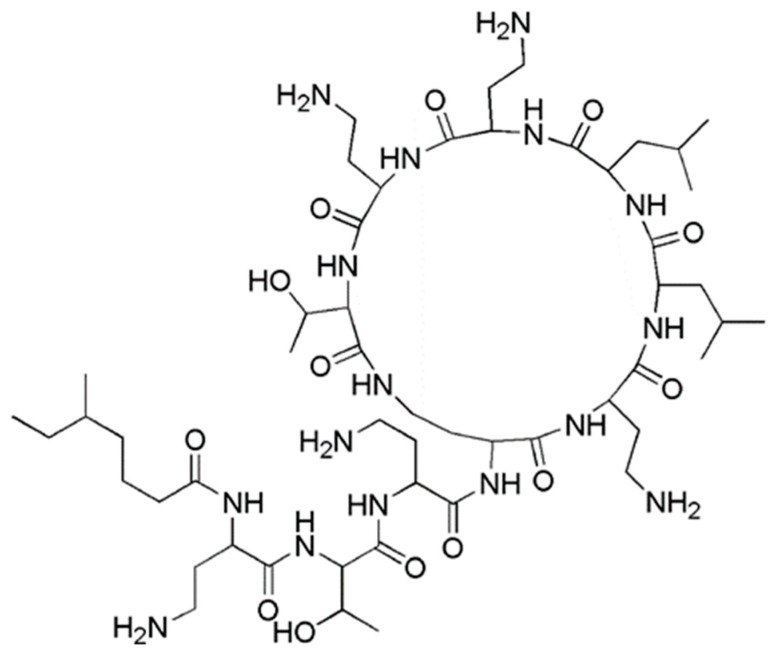
Structure of colistin.

In particular, among CAMPs, polymyxins as colistin (Figure 1) and polymyxin B, that differs by colistin only for a single amino acid in the peptide ring [14], are cyclic polypeptides produced by some strains of *Bacillus polymyxa*, specific to counteract Gram-negative bacteria that nowadays are highly critical for human health. In fact, polymyxins, although totally ineffective on Gram-positive bacteria [14], are highly active against most members of Gram-negative strains, including the Enterobacteriaceae family, counting *E. coli, Enterobacter* spp.*, Klebsiella* spp.*, Citrobacter* spp.*, Salmonella* spp. and *Shigella* spp. and common non fermentative Gram-negative bacteria, such as *A. baumannii, P. aeruginosa* and *Stenotrophomonas maltophilia* [14].

These molecules, differently from conventional not cationic antibiotics, thanks to their positive charge, without needing to enter the bacteria cell and interfere with specific metabolic processes, act with a rapid and non-specific disruptive action on bacteria membranes and kill pathogens simply on contact, before they manage to organize adaptive processes for becoming resistant. Unfortunately, despite their considerable activity, the massive clinical application of native CAMPs, as well as of polymyxins, is hampered by their poor stability, high costs of production and strong toxicity for human cells.

Assuming that the cation character can represent a fundamental characteristic for manufacturing antimicrobial devices active where old molecules fail, in the recent years, starting from natural CAMPs, taken as template molecules, the scientists have endeavored to develop less toxic and more low-cost mimics of CAMPs.

Synthetic cationic peptides, natural and synthetic cationic polymers and positively charged dendrimers were proposed, to be used as novel and unconventional antimicrobial devices with potential to counteract infections by multidrug resistant Gram-negative strains [15,16,17,18,19].

Among the developed mimic of CAMPs, cationic antimicrobials in the form of macromolecules have gained increasing attention by the scientific community because an antimicrobial polymer if compared to small drug molecules could be endowed with several advantages, such as more long-term activity, limited residual toxicity, chemical stability, non-volatility and incapacity to permeate through the skin thanks to its macromolecular structure and high molecular weight (MW) [20,21].

**Figure 2:**
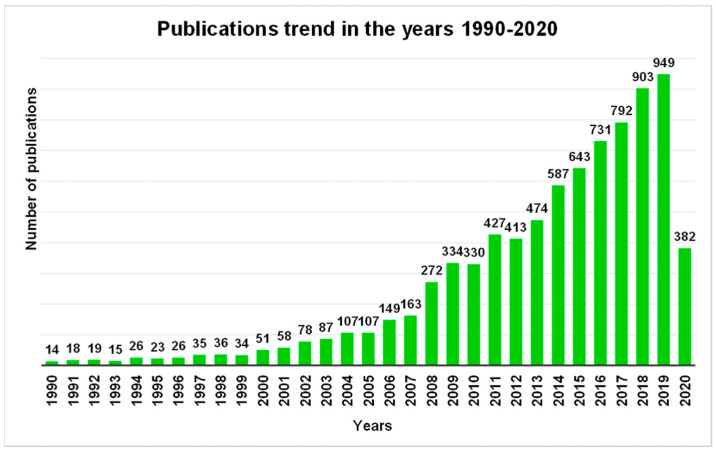
Number of publications as a function of time that contain the phrase “antimicrobial polymer” via Scopus. These data include the cationic antimicrobial polymers literature (the scope of this review).

In the last decades, antimicrobial polymers have aroused increasing interest among scientific community until becoming a “hot” topic as confirmed and highlighted also by the publications trend in the years 1990–2020 (Figure 2).

The graph in Figure 2 definitely emphasizes how over 30 years, the scientific production and therefore the research in the field of antimicrobial polymers went from being very limited until 2000, to growing steadily until it assumed an exponential increase in the last decade, probably hand in hand to how the concern for the dangers represented by multidrug-resistant Gram-negative bacteria has grown.

On this background, in this work, the most important achievements in the field of cationic antimicrobial polymers (CAPs) were reviewed. An updated information concerning the different types of the industrialized CAPs active on Gram-negative bacteria that are highly critical for human health, their structures, the supposed mechanism of action and their uses or field of applications, were reported. In order not to re-propose a simple update of other reviews already available, the most part of information was provided in the form of tables, a more “readers-friendly” tool, which allows faster consultation and quicker learning of the essential characteristics of the various antimicrobial agents herein discussed.

## 2. An Overview on CAMPs, the Template Molecules that Inspired the Development of Cationic Antimicrobial Devices

CAMPs are a class of cationic peptides active on Gram-positive, Gram-negative bacteria, fungi, protozoa and yeast.

Even if the exact mechanism of action of CAMPs is continuously under debate, the assumption recognized for long time asserts that concerning Gram-negative bacteria, thanks to their cationic structure, CAMPs first, interact with the anionic constituents of the outer membrane (OM), as LPS and phospholipids [22].

In particular, it was reported that concerning polymyxins, the *α,γ*-diaminobutyric acid (Dab) residue of the positively charged antimicrobial compounds interacts with the phosphate groups of the negatively charged lipid A, present in the LPS in OM. The stabilizing divalent cations, Ca^2+^ and Mg^2+^ are then displaced from the negatively charged phosphate groups of membrane lipids and consequently LPS is destabilized [14].

By these events, CAMPs cause the OM permeabilization, induce impairments in its integrity and provoke pores formation. The increased permeability of OM allows CAMPs to reach the inner cytoplasmic membrane (CM), to interact with its phospholipids, to cause CM increasing permeabilization, thus leading to leakage of the cytoplasmic content and to cell death [22,23,24]. Summing up, CAMPs act by a “brute-force action” based on a non-specific mechanism, factor that make resistance less likely to develop [25,26].

Differently from other antibiotics, CAMPs do not need to cross the CM and enter the cell, to neutralize bacteria.

The permeabilization of the bacteria membrane by CAMPs action, can be exploited in synergistic therapies for allowing the associated antibiotic to easily enter the bacterial cell and to reach higher concentrations inside the cell at low dosage of administration, thus reducing systemic toxicity [27].

Anyway, more specific and targeted mechanisms of action of CAMPs and polymyxins were also reported, such as the interfering activity with central cellular processes, such as DNA and protein syntheses, protein folding and cell wall synthesis [28,29].

Moreover, polymyxins possess the so called “endotoxin effect”. In Gram-negative bacteria, the endotoxin is the lipid A, which is a portion of the LPS and polymyxins have the ability to bind this endotoxin, thus neutralizing LPS molecules, which will be released during cell lysis [14].

Furthermore, polymyxins inhibit the vital respiratory enzymes, as type II nicotinamide adenine dinucleotide-quinone oxidoreductases [NDH-2] present in the bacterial CM [14].

Unfortunately, native CAMPs lack specificity and may interact without distinction also with the membrane of eukaryotic (mammalian) cells, with preference for red blood cells (RBCs), causing hemolysis and RBCs death, if used for systemic treatment [30].

Poor biocompatibility and hemolytic toxicity may be addressed by chemical modifications, voted to reduce CAMPs cationic character, but mitigation of cytotoxicity often translated in a reduction of effectiveness.

In this regard, in a successful study by Jiang et al. (2014), it was reported the substitution of positively charged residue(s) in the center of the nonpolar face of amphipathic *α*-helical or cyclic *β*-sheet of piscidin 1, a CAMP isolated from fish and dermaseptin S4, isolated from frog, with one or two lysine residue(s) [29]. By this strategy, a selectivity between eukaryotic and prokaryotic membranes was achieved, while the antimicrobial activity was maintained and the hemolytic activity and cell toxicity to mammalian cells was decreased or nullified [30].

The total inactivity of polymyxins on Gram-positive bacteria and the higher efficiency *versus* Gram-negative bacteria usually showed by CAMPs, depend on the different composition of the membranes of the two types of bacteria and on the main mechanism of action of CAMPs, involving electrostatic interactions with bacterial membranes. If compared to Gram-positive ones, Gram-negative bacteria own a more complex cell wall, made of two negative phospholipidic membranes and a surface characterized by a higher density of negative charge, due to the presence of lipopolysaccharide (LPS), encompassing phosphate and pyrophosphate groups, in the outer membrane (OM). On the contrary, the negative charge of the unique membrane of Gram-positive bacteria is due only to phospholipids. LPS is absent and peptidoglycan, polysaccharides and teichoic acids are the other constituents. Therefore, since CAMPs are amphipathic molecules typically positively charged [31], are absorbed easier and with stronger electrostatic interactions, which are the first events in the pathway that leads to bacteria death, on Gram-negative bacteria, rather than on Gram-positive ones. Unfortunately, despite their considerable activity against multi drug-resistant Gram-negative bacteria, the clinical application of native CAMPs is limited by their strong toxicity for human cells.

As examples, the use of nisin (Figure 2), which is a polycyclic peptide produced by *Lactococcus lactis* bacterium, is restricted to food industry as antimicrobial preservative, while the use of colistin, after an extensive clinical application for counteracting severe infections from Gram-negative bacteria, starting from 1970s, was delimited to ophthalmic and topical uses, because of its nephrotoxicity [14]. Systemic or nebulized colistin was continued only for cystic fibrosis patients [14] and its parenteral administration was adopted as last-resort for alarming infections by multidrug-resistant (MDR) Gram-negative, such as pneumonia [32].

**Figure 3:**
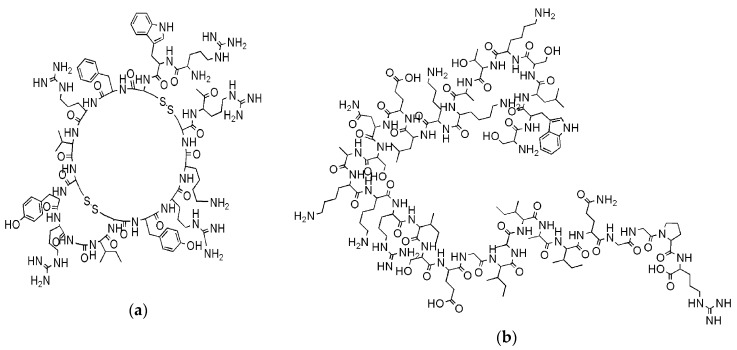
Examples of cationic antimicrobial peptides (CAMPs) not susceptible to develop resistance: (**a**) Structure of tachyplesin II; (**b**) structure of cecropin P1.

For years, it was replaced by innovative and less toxic aminoglycosides, quinolones and *β*-lactams, but the increasing incidence of MDR Gram-negative bacteria has coerced scientists to reconsider systemic polymyxins, since currently are often the only available antibiotic agents effective against MDR organisms, as the carbapenemase-producing bacteria [14]. Although it seems less probable to occur than for classical antibiotics, the probable development of bacterial resistance to CAMPs is an additional distress associated to their massive application [25,26]. Anyway, while traditional antibiotics, in order to neutralize bacteria, generally targets three organs consists: cell wall, translation machinery and DNA replication system and each one of these modalities of acting is susceptible to bacterial resistance, CAMPs are effective also without interfering with specific metabolic processes [25]. By acting with a rapid and non-specific disruptive action on bacteria membranes, they inhibit the growth of pathogens or kill them simply on contact and before bacteria manage to organize adaptive processes for becoming resistant in time. As reported, some CAMPs, as tachyplesin II and cecropin P1 (Figure 3a,b, respectively) proved limited evolution of resistance and, antibiotic-resistant bacteria display no cross-resistance towards them [33].

Although other issues such as low peptide stability, costly production and pleiotropic biologic were raised by skeptics [26], thanks to their broad spectrum of activity and extreme rapidity in killing bacteria, compared to chemical antibiotics, much effort was made to find potential novel antibacterial drug candidates among CAMPs [22,34].

## 3. Antimicrobial Polymers

Polymers have gained increasing attention by the scientific community as promising materials to prepare antimicrobial agents because of several advantages. An antibacterial polymeric device, differently from small drug molecules, could be endowed with more long-term activity, limited residual toxicity, chemical stability, non-volatility and incapacity to permeate through the skin thanks to its macromolecular structure and high molecular weight (MW) [20,21].

The developed antimicrobial polymers can be divided into three wide families as reported in Table 1.

In the biocidal polymers the antimicrobial site of biocidal polymers is embodied by the entire macromolecule, they do not require bioactive repeating units and are necessarily cationic.

The biocidal polymers can be obtained either by the polymerization of cationic monomers not necessarily active and frequently without antibacterial activity or by functionalizing an inactive polymeric scaffold with inactive cationic groups to form a cationic macromolecule with antimicrobial activity due to its high density of positive charge. Biocidal polymers are active because they are positively charged macromolecules that, miming CAMPs, are able to kill bacteria on contact, by a disruptive action on their anionic cell membranes [35].

Differently, polymeric biocide derives from the polymerization of antibacterial monomers, which can be cationic, anionic or uncharged. Therefore, polymeric biocides are not necessarily cationic, they can also be anionic or neutral, are less active than monomers and act with the same mechanism of action as monomers. The antibacterial activity resides in the monomeric units and not in the polymer itself and in some cases, the polymer architecture can even nullify the antibacterial effects of the monomers. The mechanisms of action of polymeric biocides can therefore be of various types and do not necessarily interfere with the integrity of the bacterial membranes by destroying them. Lastly, biocide-releasing polymers consist of non-active polymers loaded with biocide moieties, covalently linked or physically entrapped, which can be released also in a targeted and/or protracted modality. In this work, it was reviewed the first class of antimicrobial polymers, i.e., the per se antimicrobial cationic polymers and in particular, those ones active on Gram-negative bacteria.

The cationic moieties possibly present in cationic antimicrobial polymers (CAPs) are in general guanidinium, tertiary sulfonium, primary, secondary, tertiary and quaternary ammonium, including also compounds containing heterocycles such as pyridine, imidazole, etc. with quaternized nitrogen atoms and quaternary phosphonium groups [36,37].

In order to obtain good materials, the architecture of the designed polymer should be stable in long-term applications, stable during the required storage time and at the conditions of its targeted application and should have a low degree of toxicity. Among the developed CAPs, few natural and semi-synthetic macromolecules and a large variety of synthetic cationic polymers were manufactured.

Since it is reasonable to think that mechanism of action of synthetic CAPs would be similar to that of CAMPs previously described and considered “membrane active agents”, the main strategies premeditated to design them, depended on the structural features of the outer envelope of the different bacterial cells. Since the scope of this work is to review CAPs active on Gram-negative bacteria, a list of the some representatives of this class was reported in Table 2, while a brief description of the cell wall of Gram-negative bacteria was provided in the subsequent Section.

## 4. Structure of Gram-negative Cells Wall

**Figure 4:**
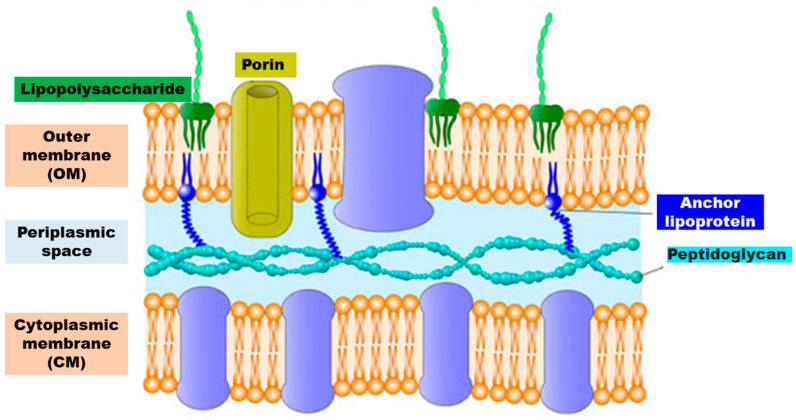
Schematic representation of the structure of the cell wall of Gram-negative bacteria.

Gram-negative outer envelope is composed of a thin peptidoglycan layer sandwiched between an inner cytoplasmic cell membrane (CM) and a bacterial outer membrane (OM) (Figure 4). The fundamental characteristics of the Gram-negative bacteria cell wall are summarized in Table 3.

As reported in Table 3, the most important characteristic of the outer envelope of the Gram-negative bacteria cells is a net negative charge, frequently stabilized by the presence of divalent cations such as Mg^2+^ and Ca^2+^. The anionic character is due to LPS present in the OM, to the phospholipids of the OM and to those of CM, which in turn is composed of a phospholipid bilayer with embedded essential functional proteins, such as enzymes. CM is semi-permeable and controls the passage of solutes and metabolites in and out of the cell cytoplasm [38].

The presence in the OM of porin channels, which slow down molecular diffusion and limit the antibacterial substances diffusion, is considered the reason of the high resistance of Gram-negative bacteria towards common antiseptics and disinfectants in comparison to Gram-positive [39].

OM and CM represent the primary target for the antibacterial agents, whose main mechanism of action mimics that of CAMPs and since they are anionic hydrophilic–hydrophobic compartments, in order to promote their absorption on bacteria, the synthetic antimicrobial polymers were mainly designed as cationic hydrophilic–hydrophobic macromolecular systems.

## 5. Antimicrobial Cationic Polymers (CAPs) and their Antibacterial “Brute-force Action”

Several combinations of hydrophilic and hydrophobic polymeric constructions were explored in order to realize the ideal CAP.

As examples, polymers with controlled MW, precise structure and composition were synthetized by advanced polymerization techniques, as reversible deactivation radical polymerization (RDRP) or reversible addition-fragmentation chain transfer polymerization (RAFT).

Block polymers, owing bi-block links, made of a hydrocarbon nonpolar hydrophobic block and of a cationic one, were developed. Furthermore, random copolymers were achieved by polymerizing a hydrophobic monomer and a hydrophilic comonomer with a functional group. Hydrophobic polynorborane-based oligomers and polymers (see Table 5 and Section 7.2) endowed with high antimicrobial effects against Gram-negative *E. coli* rather than against *S. aureus*, were synthetized.

In general, but not always, the developed CAPs are amphiphilic macromolecules and possess surface-activity properties, the adsorption/absorption ability of surfactants, high binding affinity for bacterial cells membrane and a proper lipophilicity, that allows them to cause effective damage to the structural organization and integrity of cell membranes and to lead to cell lysis [20,40,41].

In particular, CAPs like CAMPs, commonly inhibit or kill bacteria immediately on contact by causing the bacterial cell to burst, through a series of steps [20].

In particular, considering the Gram-negative bacteria of interest of this review, in the first step, the polymer adsorbs onto the OM of bacterial cell wall, in virtue of an electrostatic interaction and causes impairments, which translate in an improvement of OM permeability and pores formation. Second, the polymeric antimicrobial agent diffuses through the cell wall, adsorbs onto the CM and finally causes CM disruption. The subsequent leakage of cytoplasmic constituents including crucial cations as K^+^ leads to the death of the bacteria cell. It is evident that, while small molecule antimicrobial agents are endowed with a weak adsorption capacity and a good diffusion ability, thanks to their low MW, CAPs excel at the adsorption steps, that are crucial for disrupting CM and kill the cells (Table 4) [20].

Consequently, while the small drugs need to diffuse and enter into bacteria cells to affect vital processes, CAPs neutralize bacteria on contact without the need of interfering with more fine metabolic pathways.

Chitosan-based cationic polysaccharides [42,43], polyvinyl-based phosphonium, quaternized ammonium salts and not quaternized amine polymers [44], insoluble pyridinium-based polymers [45,46], polymers peripheral functionalized with poly(vinyl-*N*-pyridinium) salts [47,48], *ε*-poly *L*-lysine (*ε*-PL) [49], cationic amphiphilic polyacrylates [50] and branched polyethyleneimine (*b*-PEI) [51,52,53] are among others, some examples of the developed classes of natural and synthetic positively charged polymers endowed with antimicrobial properties. Table 5 reports, as far as possible complete and updated, a collection of the natural and synthetic CAPs active on Gram-negative bacteria industrialized in the last years. In particular, in the first column, the simplified structures of the positively charged polymers can be observed, while in the other columns, the target Gram-negative bacteria on which compounds were tested and their antibacterial activity is reported. Finally, the advantages and drawbacks associated to the reported compounds and their uses and/or sectors of application are also provided. For completeness of information, other details concerning the synthetic strategies and the mechanisms of action are reported in the text of Section 6 and Section 7, including also compounds not reported in Table 5. A brief description of changes caused by CAPs action in Gram-negative bacteria at molecular level and of the polymer’s structural factors that could influence their activity and toxicity are reported in Section 8 and Section 9.

## 6. Natural Positively Charged Antimicrobial Polymers

Among cationic polymers, up today, chitosan and poly(*ε*-lysine) are the only natural polymers recognized to possess antimicrobial properties [93].

### 6.1. Chitosan

Chitosan is a natural cationic polysaccharide deriving from chitin by deacetylation in basic solution and encompassing in its structure units of *β*(1 → 4)-2-amido-2-deoxy-D-glucan (D-glucosamine) and *β*(1 → 4)-acetoamido-2-deoxy-D-glucan (*N*-acetyl glucosamine) joined by glycosidic bonds [94,95,96].

Usually, commercially available contain > 75%–85% deacetylated units and have MW between 50 and 1000 kDa. The degree of deacetylation strongly influence chitosan solubility, its capacity to interact with polyanions and consequently its antimicrobial effects [97].

In addition, also MW, concentration, physical state (e.g., in solution or in solid state, as fibers, particles or films) and type of microorganism may influence chitosan activity.

Chitosan can act in two modalities, passively by provoking a reduction of protein adsorption on bacteria surface that leads to impairing the adhesion capacity of pathogens (in this case, bacteria are not killed, but only repelled) or actively, killing bacteria on contact.

However, the dominant argument is that chitosan acts principally as an external membrane disruptor, by interacting electrostatically with Gram-negative bacteria LPSs and by causing the formation of pores. Once membrane permeability is hopelessly compromised, chitosan can also behave as penetration material and, if its MW is sufficiently low (MW < 5 kD), can enter into bacteria cells, bind with microbial DNA and/or mRNA, thus interfering with transcription and translation processes.

A third supposed mechanism is based on the property of chitosan to bind metals as bivalent cations that stabilized the OM, thus favoring OM destabilization and loss of integrity.

The chitosan capacity of interacting with the negative charges from the bacterial cell surface is more effective at low pH, that allows the amine groups to be protonated, while the chelation ability is more efficient at high pH, when the positive metal ions can bound to chitosan, non-protonated amino groups and the electron pair on the amine nitrogen is available for donation to metal ions [97].

By synthetic quaternization of the nitrogen atoms of the amino groups of chitosan, semisynthetic chitosan derivatives permanently charged at any pH value were prepared [54,55].

Many methods were proposed for realizing the *N*-quaternization of the chitosan nitrogen atoms and several positively charged derivatives were prepared with different amounts of quaternary ammonium salts moieties.

They are soluble in water and have proved high antimicrobial and antibiofilm activity depending on the number of cationic groups [54,56,57,58] and can be eligible for application in pharmaceutic and biomedical fields as agents against infection by implantation of medical devices.

**Figure 5:**
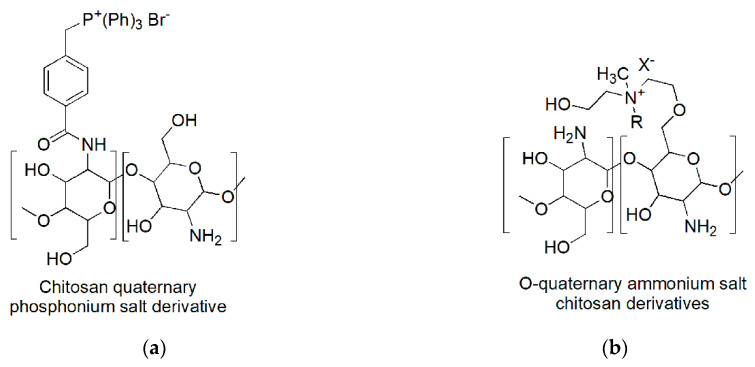
Quaternized chitosan derivatives permanently cationic: (**a**) Chitosan phosphonium salt; (**b**) *o*-quaternary chitosan ammonium salts. R: –CH_2_Ph (BNQAS–CS); –C_12_H_25_ (C_12_QAS–CS); – C_14_H_29_ (C_14_QAS–CS); – C_16_H_33_ (C_16_QAS–CS); – C_18_H_37_ (C_18_QAS–CS); X: Cl, Br.

Chitosan derivatives permanently protonated were prepared by exploiting either the amine groups or the hydroxyls to insert phosphonium or ammonium salt functions. It is the case of the chitosan derivatives used by Zhu et al. (2016) [98] (Figure 5a) and by Wang et al. (2016) (Figure 5b) [99].

*N*-quaternary phosphonium chitosan derivatives (*N*–QPCSxy) were prepared by partial amidation of NH_2_ moieties of chitosan with 4-(2,5-dioxo-pyrrolidin-1-yloxycarbonyl)-benzyl)-triphenyl-phosphonium bromides (NHS–QPS).

The best compounds achieved proved water solubility over the pH range of 3 to 12 and antibacterial activities significantly improved if compared to chitosan and low cytotoxicity. In particular, the minimum bactericidal concentration (MBC) against *E. coli* was observed at a dosage of 500 µg/mL, which allowed a red blood cell viability of 74.1% [98].

On the contrary, the chitosan chemical modification proposed by Wang et al., that led to obtain water-soluble *O*-quaternary chitosan ammonium salt (QAS–CS) bearing *N*-methyl-*N*-R-*N*-bis(2-hydroxyethyl)ammonium bromides [R =–benzyl (chloride, BNQAS–CS),–dodecyl (C_12_QAS–CS),–tetradecyl(C_14_QAS–CS),–hexadecyl(C_16_QAS–CS),–octadecyl(C_18_QAS–CS)], allowed to achieve molecules with good antibacterial abilities against Gram-positive bacteria, but bad against Gram-negative bacteria [99].

### 6.2. ε-Polylysine (ε-PL)

*ε*-PL is a cationic polyamide consisting of *L*-lysine units (n = 25–30), linked together by the *ε*-amino and the *α*-carboxyl groups. *ε*-PL toxicity is significantly lower than that of CAMPs [49] and *ε*-PL has received increasing attention in food industry as preservative additive, thanks to its strong antimicrobial effects and established safety [49]. In vivo investigations for evaluating acute oral toxicity, proved that *ε*-PL is non-toxic at the high dosage of 5 g/kg in rats [49].

Concerning its mechanism of action, Ye et al. (2013) [59] carried out studies to explain *ε*-PL antibacterial mechanism of action against *E. coli O157:H7* at the molecular level.

The results asserted that, initially the main approach is an electrostatic interaction with OM, then quickly *ε*-PL strips of the membrane, causing alteration of cytoplasm distribution, formation of pores and the onset of structural defects. The increased permeability of membrane favors bacteria penetration and DNA binding. The disruption of membrane integrity jointed to the detrimental interaction of *ε*-PL with genetic material induce oxidative stress by radical oxygen species (ROS) production and influences various gene expressions leading to bacteria death [60,61,62,63].

## 7. Synthetic Cationic Antimicrobial Polymers (CAPs)

### 7.1. Polymers Containing Quaternary Phosphonium and/or Ammonium and/or Guanidinium Groups

Polymeric quaternary ammonium salts (PQASs), quaternary phosphonium salts (PQPSs), polymeric guanidine (PGSs) and biguanidine salts (PBGSs) are classes of cationic polymer materials with high potential as antimicrobial agents, due to the high and permanent cationic character of their quaternary groups [66,75,76,100,101,102,103].

#### 7.1.1. Polymers Containing Quaternary Phosphonium and/or Ammonium Groups

PQASs and PQPSs can be obtained either through direct polymerization of monomers already containing quaternary groups, by incorporating the quaternary moieties into the previously synthesized polymers or including them by electrostatic interactions with previously synthetized sulfonate polymers.

Polymers with quaternary ammonium or phosphonium salts are materials widely explored as antimicrobial devices and have proved potent activity [104,105,106,107] and effectiveness even against bacteria that are resistant to other cationic antibacterial agents [108].

Both of them proved to be more active of the corresponding starting small molecules monomers mainly against Gram-negative bacteria with an effectiveness that resulted enhanced by the increase of polymeric chain length and by the hydrophobicity of the macromolecules [79,102,109,110].

In general, low MW antimicrobial agents including cationic monomers present several issues counting environmental toxicity and short-term antimicrobial ability. Their introduction into polymer molecules achieving biocide polymers as PQASs and PQPSs allows enhancing their efficacy and selectivity, prolonging their lifetime, minimizing the environmental problems and the residual toxicity [20].

PQASs and PQPSs, when inserted on surfaces to achieve antimicrobial surfaces able to kill airborne as well as waterborne microbes, significantly limit bacteria colonization without release of antimicrobials into the environment.

In addition, they proved higher activity of non-polymeric small antimicrobial drugs because, while their action consists in impairing the adhesion of bacteria by reducing their contact ability to the surface (without killing them), PQASs and PQPSs with quaternary ammonium or phosphonium units, commonly kill bacteria on contact [20,110,111,112,113,114].

Based on the mode of incorporation of quaternary ammonium or quaternary phosphonium monomers in the polymers, PQASs and PQPSs are classified in two categories: ionically bound or covalently attached.

Generally, the polymeric materials with active cations electrostatically bound exhibited strong antibacterial action, thanks to the release in the aqueous environment of the active cationic groups through an ion exchange mechanism. Differently, in order to exert an antimicrobial action, the class of compounds where the active cations are covalently linked, requires the contact of the polymer with the microorganisms [66].

In addition, studies of comparison between PQASs and PQPSs showed that the latter possess antimicrobial activity higher than that of polymeric quaternary ammonium salts, because of a difference of electronegativity between nitrogen and carbon atoms and phosphorous and carbon atoms [109].

The reason is attributable to the mechanism of action of the ammonium and phosphonium polymers that involves as usual, a destructive electrostatic interaction with the bacteria cell wall [66,115].

Concerning Gram-negative bacteria, the results of investigations carried with different experimental methods in the last decades, supported the hypothesis that antimicrobial polymers bearing cationic charges on the quaternary ammonium/phosphonium groups, kill bacteria by electrostatic interaction with the outer membrane (OM) and cytoplasmic membrane (CM), followed by their damage, cell lysis with release of crucial ions such as potassium [65].

Due to the difference of electronegativity existing between nitrogen and phosphorous atoms and the adjacent carbons, in ammonium cation, nitrogen exhibits a negative charge, while in phosphonium, phosphorous owns a positive charge [116].

Consequently, the stronger polarization and positive charge of phosphorous atoms in PQPSs favor easier interactions with bacteria wall thus resulting in higher effectiveness [117]. A positively charged antimicrobial random co-polymer encompassing both kinds of cationic groups was synthetized, via free radical polymerization (FRP) of acrylamide (AM), diallyl dimethyl ammonium chloride (DADMAC) and (4-penten-1-yl) triphenylphosphonium bromide (PTBT).

The obtained poly(PTPB-r-AM-r-DADMAC) copolymers, different for the content of PTBT, in addition to possess antiviral activity, proved to be effective against *E. coli* but only when the content in phosphonium monomer was higher than 49%, confirming the higher effectiveness of cation phosphonium [68].

Later, with a similar procedure, tri-blocks copolymers containing both phosphonium and ammonium groups were synthetized by AM, tributyl(4-vinylbenzyl)phosphonium (QPM) and [2-(acryloyloxy)ethyltrimethylammonium chloride (ATC) and were tested for antimicrobial and antiviral activities.

The results from antibacterial evaluations on *S. aureus* and *E. coli* and from viricidal investigations on influenza virus and adenovirus, demonstrated an excellent antibacterial activity *versus* both Gram-positive and Gram-negative bacteria and antiviral activity *versus* both enveloped and non-enveloped viruses [67].

In a study by Kougia et al. (2015), a library of homopolymers and copolymers with quaternary cationic groups either electrostatically bound or covalently linked were prepared. Furthermore, copolymers, obtained by copolymerizing the cationic monomer vinylbenzyl dimethylhexadecylammonium chloride (VBCHAM) and either hydrophilic or hydrophobic comonomers, were synthetized. Even if phosphonium co-polymers were also investigated, the study mainly focused on quaternary ammonium polymers. The antimicrobial activity, determined as a function of the contact time at 4 °C and 22 °C was evaluated against *P. aeruginosa*, *E. coli*, *S. aureus* and *Enterococcus fecalis* and eventual relationship between polymer chemical structure and antimicrobial activity was investigated and discussed [66].

VBCHAM-based copolymers in which acrylic acid (AA) was used as the comonomer and copolymers presenting both covalently attached and electrostatically bound quaternary ammonium groups showed the highest antimicrobial activity [66].

In particular, seven cationic macromolecules from this study named poly(cetyltrimethylammonium-4-styrene)sulfonate (PSSAmC_16_), poly(cetyltrimethylphosphonium-4-styrene)sulfonate (PSSPhC_16_), poly(VBCHAM), poly(methymetacrylate-co-VBCHAM) [P(MMA-co-VBCHAM)], poly(cetyltrimethylammonium-4-styrene) sodium sulfonate-co-VBCHAM) [P(SSNa-co-VBCHAM)], poly(acrylamide-co-VBCHAM) [P(AA-co-VBCHAM)] and poly(cetyltrimethylammonium-4-styrene)sulfonate-co-VBCHAM) [P(SSAmC_16_-co-VBCHAM)] were reported in Table 5 and the antimicrobial activity of each one against Gram-negative bacteria were provided.

In order to evaluate practical applications of developed CAPs, it was investigated if their antimicrobial activity could be maintained when they are embodied in polysulfone (PSF), a polymer typically used in medical devices. For this experiment, the copolymers P(AA-co-VBCHAM) and P(SSAmC_16_-co-VBCHAM) and the homopolymer PSSAmC_16_, which were the most active, were entrapped in PSF and the CAPs-enriched PSF-based membrane were essayed. The results confirmed that the developed antimicrobial materials remain remarkably efficient even when they are incorporated in PSF membranes [66].

#### 7.1.2. Polymers Containing Quaternary Guanidinium Groups

Among biguanidinium polymers, poly(hexamethylene biguanide chloride) (PHMB) was the first antimicrobial polymer whose mechanism of interaction with phospholipid membranes was studied by Broxton and coworkers on *E. coli* [77,118].

In particular, it was observed, that the sequence of events during PHMB interaction with the cell envelope of *E. coli* involves first, a rapid attraction of PHMB toward the negatively charged bacterial cell surface, thanks to strong and specific adsorption to phosphate groups of compounds present in OM. As a consequence, the integrity of the OM is impaired and PHMB is allowed to proceed and to be attracted to the inner CM, where an additional binding of PHMB to phospholipids occurs. Consequently, also the integrity of inner membrane begin to impair and if PHMB concentrations are low, the increment of membrane permeability causes only the loss of potassium ions (K^+^) and provokes bacterial stasis. Progressively, higher concentrations of PHMB, increases the extent of the damage and the size of pores, which allow the loss of larger inorganic species as Cs ^+^, Na ^+^, Li ^+^ and inorganic phosphate. This event leads to a complete loss of membrane functionalities with leak of other essential cellular components, precipitation of intracellular constituents and bacterial death [77].

The damage to the CM by PHMB is non-specific, immediate and irreversible. Practically, a scenario similar to that observed for polyvinyl benzyl dimethyl butyl ammonium chloride [65,75].

In regard of growth inhibitory activity and bactericidal activity, PHMB with high MW, i.e., n ≥ 10, proved to be effective at very low value of MIC and MBC (Table 5), PHMB with 2 > n < 10, proved good activity (Table 5) while the activity of low MW dimers was questionable. It failed to inhibit motility in actively growing cultures and did not totally inhibit growth [77].

In a study by Ikeda et al. (1984), it was aroused a concern about the exact evaluation of antimicrobial activity of biguanidinium compounds in culture medium, because of interfering interactions between the polymeric biguanides and some culture medium constituents.

In this regard, acrylate monomers with pendant biguanide groups were successfully synthesized and their homopolymers and copolymers with acrylamide were prepared by radical polymerization using AIBN as initiator. Evaluated in a clean system, these CAPs showed to be higher effective against Gram-positive bacteria, rather than on Gram-negative strains, but to be much more active than the monomeric species [15,65].

Synthetic guanidinium and biguanidinium antimicrobial polymers endowed with a proper amphiphilic balance, which allows high selectivity for bacteria and good antimicrobial activity, are considered the best mimics of CAMPs.

In this regard, polyhexamethylene guanidine hydrochloride (PHMG) and three its analogs, i.e., polybutamethylene guanidine hydrochloride, polyoctamethylene guanidine hydrochloride (POMG) and poly(m-xylylene) guanidine hydrochloride, were prepared by reacting guanidine hydrochloride with the proper di-alkyl amine. The antimicrobial properties of the obtained polymers were investigated on 370 clinical strains, often involved in nosocomial infections, 96 isolates of which were antibiotics-resistant. MIC values and MBC data obtained through the time killing essay were measured and were reported in Table 5 [78].

The best compound was POMG, that provided MIC values (0.5–16 µg/mL) even lower than those of chlorhexidine digluconate (2–64 µg/mL) against all the 370 antibiotics-susceptible and antibiotics-resistant clinical strains. Concerning the interest of the present review, POMG displayed excellent activity (2–16 µg/mL) against several representative of Gram-negative bacteria (Table 5) [78].

The killing curves showed that POMG was bactericidal at 5 µg/mL, concentration that caused an approximate 6 log_10_ reduction in the numbers of CFU for clinically isolated *P. aeruginosa* at 4 h [78].

The broad activity of POMG against antibiotic-resistant bacteria suggests that cationic guanidine-based polymers possess high potential for the development of novel potent antimicrobials for clinical applications.

Copolymers with functionalized guanidine pendant groups were prepared by RAFT polymerization and were evaluated for their antimicrobial activity against some Gram-positive strains, *C. albicans* and *E. coli*, as well as for the hemolytic toxicity [69,74]. While antimicrobial activity *versus* Gram-positive bacteria and antifungal effects resulted considerable, antibacterial activity on *E. coli* was debatable.

### 7.2. Polynorborane-based Antimicrobial Polymers

Designed just to mimic CAMPs, polynorboranes (PNBs)-based antimicrobial polymers possess an amphiphilic structure characterized by having the cationic hydrophilic fragment segregated onto one region (or face) of the macromolecule and the hydrophobic portion, usually constituted by hydrocarbon chains, distinctly onto the opposite face.

In this regard, such polymers are called “facially amphiphilic” (FA) and were synthetized by polymerizing FA norbornene-based monomers, with different protonated groups, such as primary amine, guanidine or pyridine, located on a side alkyl chain, pending from the nitrogen atom of the bicyclic norbornane structure (Table 5).

The mechanism of action of these polymers, involves as usually, an initial interaction with OM, the creation of pores, the insertion of the biocide into the bacteria cell wall, a second electrostatic interaction with CM, the impairment of its integrity, the progressive increase of its permeability up to its disruption, loss of cytoplasmic material and bacteria death. The type of counterions, the length of alkyl side chains and also the molecular charge density strongly influence the activity and the selectivity of PNBs polymers.

Thanks to their amphiphilic structure, antimicrobial PNBs possess particular ability in inserting and disrupting the CM of bacteria.

Alkyl hydrophobic norbornene-type polymers and the analogous oxanorbornene-based hydrophilic macromolecules, containing primary alkyl ammonium groups as cationic moieties were prepared by Ilker et al. (2004) [86]. The first ones, although very active *versus* representatives of Gram-negative bacteria, proved to be not selective for pathogens, thus resulting considerably toxic on mammalian cells, as established by the vesicle-dye leakage assays (Table 5). On the contrary, the latter were less cytotoxic, but practically inactive [86].

By random copolymerization of two types of alkyl hydrophobic norbornene monomers, it was possible to tune the overall hydrophobicity of the polymer achieving CAPs with high selectivity (>100) and considerable activity against *E. coli* (MIC [µg/mL, (µΜ)] = 40, 2.6–3.3).

Similar results were obtained later by Gabriel et al. (2009), for slightly modified oxanorbornene-based hydrophilic polymers that proved to be endowed with low cytotoxicity and good selectivity, but were practically inactive (Table 5) [85].

Later a good solution, in terms of preparing compounds with high antimicrobial activity on bacteria and low hemolytic toxicity on human cells, was to replace the primary ammonium group onto the side alkyl chain with the guanidinium one [87].

In this regard, a polyguanidinium oxanorbornene (PGON) compound was synthesized from norbornene monomers via ring-opening metathesis polymerization (ROMP), which in time killing studies proved to be lethal for bacteria and not only bacteriostatic (Table 5) [87].

A broad library of highly active antimicrobial FA oxanorbornene monomers were prepared and after ROMP and deprotection, provided several series of polynorbornene-derived polymers with tunable activity and selectivity [119]. Polyamine oxanorbornene-based antimicrobial random copolymers, with high hydrophobicity were prepared by performing two different approaches. One strategy involved the copolymerization of two hydrophobic FA monomers with cationic primary ammonium groups on side alkyl chains, while the other consisted in copolymerizing one cationic primary ammonium oxanorbornene monomer and a hydrophobic alkyl amine oxanorbornene comonomer.

By following the second strategy, a series of copolymers endowed with significant antibacterial activity and tunable selectivity were prepared [85].

Amphiphilic polyoxanorbornene-based polymers having different quaternary alkyl pyridinium side chains were synthesized by Eren et al. (2008), but with questionable success [88].

Compounds with a C_4_ side chain or shorter proved low antimicrobial activity and low hemolytic toxicity on human red blood cells, while compounds with a side chain longer than C_6_ proved high antimicrobial effect, but low selectivity for bacterial over mammalian cells [88].

### 7.3. Polymers Containing not Quaternized Amine Groups

For years, it was thought that fixing permanent cationic charges on polymers by quaternization of amine or phosphorus groups could be the best way to achieve polymers with enhanced antimicrobial effects. To disprove this belief, polymer systems encompassing not quaternary protonated amine groups were synthetized and their antimicrobial activity was evaluated and compared to that of *N*-quaternized analogous derivatives.

In this regard, polystyrene-based polymers, containing tertiary amine groups susceptible of reversible protonation, exerted bactericidal activity similar to that of the peptide toxin melittin and somewhat lower activity than that of a potent derivative of the host defense peptide known as magainin II [71].

For clarity, host defense peptide is another broader term to call CAMPs, which takes into account, that small cationic amphipathic peptides have strong potential not only as antimicrobials, but also as antibiofilm agents, immune modulators and anti-inflammatories [120].

The not quaternary compounds, compared to the permanently cationic corresponding *N*-quaternized macromolecules, showed far higher antimicrobial activity, suggesting that reversible *N*-protonation leads to greater biocidal activity than irreversible *N*-quaternization [71].

Unfortunately, protonable amine polymers, not exerting their antibacterial activity by a detergent like membrane disruption mechanism, lacked the selectivity of magainin II and showed high hemolytic toxicity, mimicking the not selective melittin behavior [71].

Amphiphilic methacrylamide random copolymers, bearing reversibly protonated primary or tertiary amine groups and encompassing a hydrocarbon hydrophobic side chains, were prepared and their antimicrobial and hemolytic properties were compared with those of similar macromolecules, containing quaternary ammonium groups [72].

The not quaternized copolymers owing the primary amine groups proved considerable antimicrobial activity on *E. coli* by a membrane-disrupting action [72] and were tunable in order to achieve CAPs with considerable antimicrobial activity and low hemolytic toxicity (Table 5). Concerning this, Palermo et al. (2009) demonstrated that antimicrobial activities and biocompatibility depend in a different manner on the mole fraction of the alkyl side chains, on the length of alkyl groups and on ionic charge density [121].

As examples, dense cationic charge leads to cytotoxicity, whereas excessive hydrophobicity leads to hemolysis associated to higher antimicrobial activity and a careful balance of structural features is necessary for achieving a well-performant antimicrobial device with low level of toxicity.

Analogs macromolecules containing tertiary amine groups proved minor antimicrobial activity by 100 times and less selectivity, while the quaternized co-polymers, in order to exert acceptable antimicrobial activity required a greater amount of hydrophobic comonomer and therefore showed poor selectivity and high hemolytic toxicity [72].

Water-soluble poly(diallylamines) (PDAAs) with cationic charges, thanks to the presence of pyrrolidine links with secondary or tertiary amine groups, protonated with trifluoroacetic acid, revealed potent antimicrobial activity against a representative set of bacteria and *versus Candida albicans* [79].

In particular, the less active poly(diallylammonium trifluoroacetate) (PDAATFA) derivative with MW = 24 kDa was bactericidal at 125 μg/mL and bacteriostatic at 62 μg/mL concentrations *versus E. coli* at all the conditions adopted for the experiments [79,80].

The analogs tertiary poly(diallylmethylammonium trifluoroacetate) (PDAMATFA) proved to be bactericidal *versus E. coli* even at the lower concentration of 62 μg/mL. PDAATFA and PDAMATFA derivatives with higher MW (62 kDa and 55 kDa, respectively) proved to be bactericidal also against *P. Aeruginosa, P. Mirabilis* and *K. Pneumoniae* (Table 5) [80,81].

According to what reported in 2009, without however presenting numeric data as proof, the quaternary hydrophobic polymers of this series and in particular poly(diallyldimethylammonium chloride) (namely PDADMAC in the cited work) would own week antimicrobial activity [79].

On the contrary, more recent studies showed that PDADMAC, differently named PDDA (poly (diallyldimethyl) ammonium chloride), displayed the capability of reducing of CFU counting to one of *P. Aeruginosa MDR* and *K. Pneumoniae KPC+* at minimal concentrations of 1.5 and 0.9 µg/mL, respectively [81] and excellent microbicidal action against *E. coli ATCC 25,922* (5 µg/mL) and *P. Aeruginosa* (2 µg/mL) at dosage where hemolysis was 0% [80,82,83,84,122].

The antimicrobial activity against Gram-negative bacteria of PDAA series increases with MW and with the hydrophobic-hydrophilic balance of the cationic polymers [80].

In a study by Yang et al. (2014), it was examined whether by converting the hydrophobic moiety of a synthetic antimicrobial peptide (SAMP) into a hydrophilic one could provide hydrophilic cationic polymer compounds with maintained antimicrobial activity, but enhanced biocompatibility and selectivity for bacteria cells [73].

In this regard, not quaternary primary ammonium trifluoroacetate copolymers (SAMPs) were prepared from *N*-(*tert*-butoxycarbonyl)aminoethyl methacrylate and butyl methacrylate. Then, by replacing butyl methacrylate with 2-hydroxyethyl methacrylate (HEMA), hydrophilic cationic mutants of previously prepared SAMPs were obtained.

The reactions were performed via AIBN-initiated free radical copolymerization or via RAFT copolymerization. The so obtained BOC-protected copolymers, after deprotection with trifluoroacetic acid provided the copolymer products [73]. Antibacterial assays showed that long hydrophilic-and-cationic mutants of SAMPs were membrane active against bacteria but showed strikingly reduced hemolytic toxicity and drastically enhanced selectivity [69,73].

Polymers, encompassing both tertiary amines groups protonable in a reversible way and permanent protonated azetidinium moieties, were prepared by a simple two steps procedure [70]. Briefly, waterborne multifunctional poly(vinylamine)s were first, prepared modifying commercial poly(vinyl amine), through a reaction with functional cationic couplers, in order to improve its hydrophobicity. Second, the modified poly(vinyl amine)s were furtherly functionalized by reaction with a bifunctional coupler, thus inserting azetidinium groups and alkyl chains.

A library of cationic polymer compounds was achieved, whose structure-activity relations, antimicrobial activities against Gram-positive and Gram-negative bacteria and hemolytic toxicity were determined.

Finally, the best polymer was used to prepare antimicrobial cotton surfaces, which were tested on *E. coli* establishing a 99.9% bacterial growth inhibition [70].

### 7.4. Polymers Containing Sulfonium Groups

Cationic polymers bearing sulfonium groups are similar to quaternary ammonium materials in terms of charge, but few studies were performed for evaluating their antibacterial and/or hemolytic activity.

In this regard, a study performed in 1990s reports the synthesis of poly(*p-*vinylbenzyl tetramethylene sulfonium tetrafluoroborate salts with different MW values and the assessment of their biocidal activity against *S. aureus* and *E. coli* in comparison to those of the corresponding monomer [123].

The low MW monomer showed no activity against both the Gram-positive and Gram-negative bacteria, while even if practically ineffective against *E. coli*, the polymer macromolecules, exhibited acceptable antimicrobial activity, increasing with the increase of the MW, *versus S. aureus*.

In particular, the best performant polymer (MW = 46,800) was able to kill all the bacterial cells within 30 min at the concentrations of 100 and 10 µg/mL and was capable of destroying more than 99.9% of *S. aureus* cells at the lowest concentration of 1 µg/mL within 120 min of contact [123].

The sequence of events in the mode of action of sulfonium tetrafluoroborate polymers matched the common mode of cationic biocides, which involves a phase of adsorption onto the bacterial OM, followed by impairments of membrane integrity and diffusion through the cell wall.

A second phase of binding to the CM, followed by its disruption and release of cytoplasmic constituents such as K^+^ ions, DNA and RNA up to cell death, follows.

As far as our knowledge allows, only another study dealing with sulfonium compounds with antimicrobial activity was reported. Although it does not deal with polymeric materials but concerns a library of 14 not polymeric low MW sulfonium salts, it has however reported. The prepared sulfonium salts were evaluated both for their antimicrobial activity and biocompatibility and the results showed that the major part of sulfonium salts proved higher biocompatibility and lower toxicity than those of the less toxic compound among the ammonium and phosphonium salts, taken as references. Concerning antimicrobial activity, the well performant compound was more active against *S. aureus* and *B. subtilis* by 3–4 times if compared to the best ammonium and/or phosphonium salts, but less active *versus E. coli* and *P. aeruginosa* by 5–8 times. Although sulfonium salts could be advisable as highly biocompatible devices to counteract infections by Gram-positive bacteria, their clinical applications are limited by their low thermal stability [124].

In this regard, the author suggest that the conversion of the best representatives of the reported library in polymeric compounds could be an idea for both improving antibacterial effects and enhancing stability of the small molecules.

### 7.5. Polymers Containing Heterocycles with Permanently Cationic Quaternized Nitrogen Atoms

A series of synthetic biodegradable polycarbonates containing propyl and hexyl halogenated side chains were prepared via ring-opening polymerization (ROP) performed under an inert atmosphere in a nitrogen-filled glovebox. Subsequently, they were quaternized under ambient laboratory conditions with different heterocycles, such as methyl, ethyl and butyl imidazoles and pyridines. Alkyl imidazole, as well as pyridine and dimethylamine pyridine (DMAP) polymer derivatives were achieved and were investigated concerning their antimicrobial activity (MIC) against *S. aureus, E. coli, P. aeruginosa* and *C. albicans* (fungus) [89].

In addition, hemolytic cytotoxicity (HC_50_) were assessed and the results were compared with those from analogous polymer scaffolds quaternized with trimethylamine (TMA). All compounds, TMA derivatives included, showed very low hemolytic toxicity, but concerning antimicrobial activity, heterocyclic compounds proved higher activity than TMA macromolecules, especially against *S. aureus*.

However, remaining within the interests of this review, heterocyclic polymers proved higher activity against *E. coli* and lower on *P. Aeruginosa* and hexyl derivatives both containing pyridine, DMAP or alkyl-imidazole groups were the best performants and the MIC observed against *E. coli* were < 4 µg/mL and in the range 31–63 and 8–31 µg/mL, respectively (Table 5) [89].

Studies concerning the mechanisms of action performed on *E. coli* confirmed the usual a-specific disruptive action on bacterial cell membranes that assure a minor tendency to develop drugs resistance.

A copolymer of 4-vinylpyridine (4VP), styrene (St) and divinylbenzene (DVB), namely P(4VP-St-DVB), was prepared by suspension polymerization and subsequently was quaternized with excesses of halohydrocarbons (RX) such as benzyl bromide (BzBr), C_4_H_9_Cl, C_4_H_9_Br and C_4_H_9_I to prepare series of insoluble pyridinium-type polymers, namely Q-P(4VP-St-DVB)-RX [46].

Living and death cells of *E. coli*, suspended in sterilized and distilled water, were the selected candidates on which the capability of the prepared pyridinium CAPs of interacting and adhering to bacteria cells wall was investigated, while living cells were used to evaluate their antimicrobial properties by a colony count method [46].

The results showed that, except for the compound containing iodine, insoluble pyridinium-type polymers, were capable to imprison both living and death bacterial cells by a partially irreversible adsorption or adhesion process, without killing the living cells. From these results, the cationic polymers developed could be advisable for the treatment of waste waters [46].

A number of polymers, such as high-density polyethylene (HDPE), low-density poly ethylene (LDPE), polypropylene (PP), nylon 6/6 and poly(ethylene terephthalate) (PET) were functionalized with poly(vinyl-*N*-hexyl pyridinium bromide) (hexyl-PVP) obtaining quaternary pyridinium antimicrobial surfaces whose antibacterial effects were essayed on *S. aureus* and *E. coli*.

Suspensions of bacteria in distilled water were sprayed on the hexyl-PVP-modified polymer slides, to simulate airborne bacteria. The slides were incubated overnight and the results from the counting of survived colonies showed that all polymers provided high bactericidal activity on contact, managing to kill up to 99% of bacteria [47].

The proposed methodology is eligible to render numerous products bactericidal, with limited costs, being the surfaces renewable by periodic washings.

Previously, the same authors was reported the same idea, by functionalizing glass slides with the same hexyl-PVP and performing the same procedure. Antimicrobial quaternized pyridinium glass surfaces were obtained, that proved to be able to kill on contact the 94%, > 99%, > 99.8% and > 99% of *S. aureus* (ATCC, strain 33,807), *S. epidermidis* (wild type), *P. aeruginosa* (wild type) and *E. coli* (ZK 605), respectively [48].

Unfortunately, their extensive applications are strongly limited by their poor water solubility, low biocompatibility and high risk of skin irritation. In order to address these issues, it was reported the preparation of more hydrophilic methacrylate-based copolymers with bactericidal activity and biocompatibility higher than those of quaternized poly(vinylpyridine) [90].

As comonomers were used either biocompatible HEMA and poly(ethylene glycol) methyl ether methacrylate (PEGMA). First, copolymers with different content of comonomers were prepared starting from vinylpyridine, by radical polymerization.

Second, the copolymers were quaternized with hexylbromide.

Pathogenic *E. coli* (O157:H7) was treated with the copolymers, in order to assess their antimicrobial effects and the results showed that several of the copolymers possessed antibacterial activity ∼20 times greater than that of pure quaternized poly(vinylpyridine) homopolymer. Even if the evidences of the study supported the hypothesis of good biocompatibility, the authors made no claim as to the biocompatibility of these materials [90].

In the same year, Allison et al. (2007) studied the biocompatibility of analogous pyridinium co-polymers by interaction with human red blood cells, to analyze hemolysis. The results showed that blood compatibility does not depend on the length of PEG chain in copolymers containing PEGMA. A critical weight ratio PEGMA/VP was determined which divide copolymers with no-hemolysis activity from those with 100% hemolysis [125].

Later, the hemolytic cytotoxicity expressed as HC_50_, the minimum bactericidal concentrations (MBC) determined by the ability of the antimicrobial materials to kill 10^6^ colonies of *E. coli O157* and the selectivity for some of the quaternized poly(vinylpyridine) and poly(ethylene glycol) methyl ether methacrylate copolymers, at different content of vinylpyridine (VP) [P(VP-co-PEGMA 1100)-HB] were investigated [126].

In addition, biocompatibility was evaluated by cell viability assays performed on human intestinal epithelial cells cultivated in vitro, that offer specific advantages over red blood cells (RBC) hemolysis assays, as a measure of biocompatibility of these copolymers.

The results confirmed acceptable MBC values (70 µg/mL), associated to very low HC_50_ (10,000 µg/mL) and high cells viability (1000 µg/mL) and therefore both low hemolytic activity and good selectivity and cells viability for P(VP-co-PEGMA 1100)-HB containing 50% VP.

Copolymers containing < 50% VP were endowed with high biocompatibility and very low HC_50,_ but were ineffective as antimicrobials, while copolymers containing 75% and 90% VP were more effective but endowed with very low biocompatibility and high EC_50_ [126].

The VP monomer was employed also to prepare poly(4-vinyl pyridine/poly(vinylidene fluoride) (P4VP/PVDF) polymeric microbeads, by the phase inversion technique [91].

PVDF was used as filler to achieve beads with proper mechanical strength. *N*-alkylation of the P4VP moieties was developed by using alkyl chains of different lengths, because known to be able to affect the antibacterial efficacy of the pyridinium-type polymers based on their number of carbon atoms [47,48].

Several P4VP/PVDF were achieved and were investigated for their antimicrobial efficacy against both bacterial and fungal spores. *E. coli* and *Aspergillus niger* were the representative pathogens of the two categories, respectively [91].

The pyridinium groups quaternized with C_4_–C_10_ alkyl chains proved the highest antimicrobial activity by a membrane disruptive action and in particular an number of beads of 0.8 wt % killed almost the 100% of pathogens within 20 min of an *E. coli* suspension of 105 CFU/mL.

A larger number of beads was necessary to kill *A. niger* spores at the same time, because of the more resistant nature of the fungal wall. The developed antimicrobial beads are highly stable and allows repeated applications, maintaining effective micro biocidal properties [91].

### 7.6. Quaternized Branched Polyethyleneimine Ammonium Salts

Polyethylenimine (PEIs) or polyaziridine are polymers in which the monomeric unit of the *N,N*-diethylamine is repeated, forming compounds encompassing amine groups and C_2_ aliphatic spacers.

PEIs can be linear, branched or dendrimeric, the first ones containing all secondary amines, while the others primary, secondary and tertiary amino groups.

They are produced on industrial scale, are commercially available and find applications in many fields, due to their positively charged character [127].

Among the several applications, PEIs are reported to be an effective permeabilizer of the OM of Gram-negative bacteria [51].

In this regard, the effect of commercial *b*-PEI (50 kDa) on the OM of representatives of Gram-negative bacteria as *E. coli, P. aeruginosa* and *S. typhimurium* was investigated by evaluating the bacterial uptake of l-*N*-phenylnaphthylamine, which is a hydrophobic probe indicating increased hydrophobic permeation of the OM. The uptake was prominent at the low concentration of 20 µg/mL. In addition, PEIs were able to sensitize the bacteria under study to the hydrophobic antibiotics as clindamycin, erythromycin, fucidin, novobiocin and rifampicin, to the lytic action of the detergent SDS and concerning *P. aeruginosa*, also to the non-ionic detergent Triton X-100. From the results, it was reported that PEIs showed to be a potent permeabilizer of the OM of Gram-negative bacteria, even if it does not inhibit the growth of bacteria to any significant extent [51].

On the contrary, alkylated quaternized PEIs attached to flat macroscopic surfaces and to nanoparticles proved high bactericidal effects toward both Gram-positive and Gram-negative pathogenic bacteria.

Concerning this, the findings regarding the antimicrobial properties of surfaces observed by immobilizing quaternized alkyl polyvinylpyridinium salts onto coating glass and plastic slides were extended to *b*-PEIs, used in place of PVP.

*b*-PEI (≥ 25 kDa) were first, attached to NH_2_-glass slides and to magnetic Fe_3_O_4_ nanoparticles containing NH_2_ groups, then were either alkylated only with an alkyl bromide derivative or furtherly methylated by reaction with iodomethane [52].

After a screening performed on *S. aureus* to detect the candidate suitable for further investigation, the hexyl-PEI glass slides and NPs were tested against other airborne *S. epidermidis, P. aeruginosa and E. coli* with promising results. The bactericidal efficiency *versus* Gram-negative bacteria of our interest was in the range 67%–74% for the devices containing only the C_1_–C_18_ alkyl chain and 95%–97% for the furtherly methylated ones (Table 5) [52].

The year later, following an analogous synthetic pathway, the same authors immobilized 750 kDa *b*-PEIs onto cotton, wool, nylon or polyester cloths and furtherly alkylated the PEIs-modified materials with ethyl and methyl chains achieving permanently cationic PEIs-based textiles. Their micro biocidal efficiencies were assessed towards airborne bacteria and fungi [53].

The promising results showed that micro biocidal efficiency against *E. coli* was in the range 96%–99%, whereas *versus P. aeruginosa* in the range 97%–98%.

Heine and co-workers reported the synthesis of two kinds of amphiphilic compounds (series B-I and B-II) and then the preparation of three kinds of amphiphilic poly(ethylene imine)s (series PEI-I, PEI-II and PEI-III) randomly linked to cationic and hydrophobic groups (PEI-I), to compounds of series B-I (PEI-II) and to compounds of series B-II (PEI-III).

In particular, compounds B-I encompass alkyl chains directly attached to the cationic group, while compounds B-II have the cationic group and the alkyl chains connected by a spacer [92].

All compounds B-I and B-II and modified PEI polymers were tested to evaluate their antibacterial properties against *Bacillus subtilis, S. aureus* and *E. coli*, while the more active were selected to investigate the hemolytic toxicity. Concerning amphiphilic compounds, the highest activity against *E. coli* of our interest, was showed by two compounds of B-I type having C_14_–C_18_ chains (MIC = 8 and 10 µg/mL), but while the most active was hemolytic at concentration far higher than MIC (EC_50_ = 22 µg/mL), the other showed an EC_50_ lower than MIC (4 µg/mL).

The most active among compounds of B-II type similarly possessed C_14_–C_18_ chains and showed a higher MIC of 20 µg/mL and an EC_50_ = 28 µg/mL.

Polymer materials were less active than amphiphilic compounds and showed higher hemolytic toxicity. On *E. coli*, PEI-II polymers were the most active (MIC of 60–100 µg/mL) but were also the most toxic for red blood cells (EC_50_ << 1 µg/mL) [92].

## 8. Molecular Changes Caused by Cationic Antimicrobial Polymers: A Study Performed

The modality of interactions between CAPs and bacterial membranes was investigated by using artificial lipid bilayers for simulating the permeability barrier of cell membranes and several linear cationic polyelectrolytes, such as ammonium polybases (polylysine, polyallylamine, poly(ethylenimine) (PEI)) and quaternary ammonium polysalts (polyionenes, quaternized poly(vinylpyridine) (PVP)). When the models of lipid membranes were exposed to cationic polymers, revealed common scenarios. After the formation of interface complexes between the negatively charged groups of lipid molecules and proteins of the membranes and free cationic groups of positively charged polymers happens, a translocation of the negatively charged molecules of lipids from the inside to the outside of the membrane (“flip–flop” effect) and a lateral segregation of the negatively charged lipids, occur [128].

In a study by Timofeeva et al. (2009), TEM images of the *E. coli* treated with aqueous solutions of a CAP, confirmed the mechanism involving membrane-disrupting action. In particular, the morphologic changes in the bacteria involved OM, CM, cytoplasm, ribosomes and nucleoid [79].

## 9. Main Factors Influencing the Antimicrobial Properties and Cytotoxic Activities of CAPs

The substantial bactericidal efficiency of a CAP can be related to the polymerization degree, to its hydrophobic mass, to the enlargement of the polymer coil and to the absolute number of active cationic centers on a single polyelectrolyte molecule.

In this contest, the cooperative properties of the cationic polymer molecule, which are responsible for strengthening its adsorption ability, binding affinity and destructive interaction with the bacteria cell, are the pivotal factors that influence its bactericidal effect.

The MW, the cationic charge density, the type of counterion, the global hydrophobicity and the hydrophilic–lipophilic balance (HLB) are the factors that profoundly impact the efficacy of many CAPs.

It was reported that an optimal MW ranges from 50 to no more than 100 kDa [79], even if in some cases the biocidal effect of some CAPs *versus* particular bacteria enhances incessantly with the growth of MW. In this regard, Ikeda et al. have indicated that adsorption ability and capability to penetrate through OM/CM are the key factors that control antimicrobial activity of CAPs [129].

In addition, the presence of alkyl groups may influence the antimicrobial efficacy of CAPs based on the length of the alkyl hydrocarbon chains. With the elongation of alkyl chains, the polymer behavior changes and, though its adsorption/absorption ability and lipophilicity result improved, variations in the HLB could modify CAP ability in counteract different microorganisms. It can be assumed, that different optimal alkyl appendage lengths for different macromolecular systems are necessary to achieve higher antimicrobial efficacy against a given type of microorganism.

Examples of variations in the antimicrobial activity of some CAPs, in function of variations in MW and alkyl chains length, were reported in Table 6.

As reported [134], the spatial separation of the positive charge and the presence of hydrophobic alkyl side chains, may influence both the antimicrobial properties and the hemolytic and cytotoxic effect to human cells. In particular, higher membrane-disrupting ability was observed when the charge is spatially separated from the alkyl chain [134].

Consequently, on one hand the antibacterial activity was improved, but on the other hand, also the hemolytic activities and toxicity on mammalian cells resulted increased.

In another study, it was reported that, the antimicrobial activity against *E. coli* and *S. aureus*, as well as the biocompatibility of methacrylamide-based random copolymers depend, in a different manner, on the mole fraction of the alkyl side chains and their length, but are strictly correlated to the content in primary amine groups, thus evidencing the membrane-disrupting action of the co-polymers [121].

In this contest, other examples of correlations between polymers structural properties and antimicrobial activity/toxicity were reported in Table 7.

As described in Table 5 and in Section 6 and Section 7, several of the antimicrobial cationic polymers developed, have proved good biocompatibility and low or even absent hemolytic toxicity. The explanation of a certain selectivity for bacteria cells resides in the different lipid composition of pathogen cell wall and of mammalian cell membranes. While bacterial membranes contain lipids with net anionic charge and no cholesterol, eukaryote membranes encompass zwitterionic lipids and cholesterol.

In particular, the membrane of eukaryotic cells is made of cholesterol (up to 25%), phosphatidylcholine lipids and an essentially zwitterionic neutral outer leaflet [137].

Differently, the membranes of Gram-negative bacterial cells do not contain cholesterol, have low levels of zwitterionic lipids as phosphatidylethanolamine and contain considerable levels of negatively charged lipids including phosphatidylglycerol, cardiolipin (20%–25%) and LPS. In this regard, cationic antimicrobials are rationally more promptly attracted by the strong negativity of bacterial OM and are readily inserted into bacterial lipid mixtures, rather than by the neutral membrane of mammalian cells [137].

Interestingly, no insertion of cationic materials was detected in the model of “mammalian” membranes used for experiments and no toxicity was observed [137].

In addition, it was established that cholesterol, which is present in mammalian cells, but absent in bacterial membranes, play a pivotal role in limiting or hampering the CAPs penetration, by acting as a “protectant” [137].

## 10. Conclusions and Perspectives

The incidence of multidrug resistant Gram-negative infections are one of the emerging problems that disturbs several human activities and life sectors such as food industry, food processing, transportation and healthcare services. Gram-negative bacteria cause infections including pneumonia, bloodstream infections, wound or surgical site infections and meningitis above all in healthcare settings. The concern associated to Gram-negative infections is destined to increase in importance, as traditional therapeutic remedies have become limited. Nowadays, Gram-negative bacteria are resistant to multiple drugs due to built-in abilities, which over the years have make them able to find new ways to be resilient and to pass genetic materials that enabled other bacteria to become drug-resistant as well. The well-known cephalosporins, fluoroquinolones and carbapenems gradually have turned out to be ineffective with the result of a worrying higher mortality rate across the world. Aggressive recommendations, if implemented, can prevent the spread of Gram-negatives, but in order to actually address this issue, it is necessary to identify probable novel lead molecules to combat these infections.

Natural cationic antimicrobial peptides (CAMPs), as polymyxins, possess high activity *versus* alarming bacteria as multi-drugs resistant Gram-negative *Klebsiella* spp.*, Acinetobacter* spp.*, P. aeruginosa and E. coli* but, in addition to be high-costly and unstable, are too toxic for human cells for being extensively used in clinical applications. On the example of CAMPs, in the last decades and up today, researchers’ efforts were and are focused on the synthesis of less toxic compounds that mimic AMPs, in terms of mechanism of action and effectiveness. Synthetic cationic compounds and antimicrobial peptides were developed and essayed with promising outcomes.

In this challenge, polymers have gained increasing attention by the scientific community, as promising materials to prepare antimicrobial agents, because differently from small drug molecules, they could be endowed with more long-term activity, limited residual toxicity, chemical stability, non-volatility and incapacity to permeate through the skin thanks to its macromolecular structure and high MW.

Starting from these observations, cationic polymeric materials encompassing the main structural features of CAMPs, mimicking their a-specific mechanism of actions, based on the disruption of OM and CM and able to kill rapidly bacteria on contact, were developed.

Although the pending issue of hemolytic toxicity and biodegradability of cationic polymeric materials is still left not completely solved, these macromolecules possess a higher affinity towards the anionic membrane of bacteria and a reduced toxicity toward eukaryotic cells, have low tendency to develop resistance and could have a decisive role in the global effort to find effective solutions for counteracting resistant Gram-negative bacteria.

However, although the scientific advances achieved during the last years are encouraging, further research work both in vitro and in vivo, is mandatory to enhance the antimicrobial activity *versus* more strains of bacteria, to increase the long-term stability, to nullify the residual toxicity, to develop cost-effective solutions and to allow a more extensive clinical application.

In the opinion of the authors, among the profuse production of CAPs, the compounds containing guanidine or bi-guanidine residues appear as the materials that best find an optimal compromise between good antimicrobial activity, low hemolytic toxicity and good biocompatibility.

Considering the performances shown by guanidinium antimicrobial polymers already prepared and bearing in mind the incomparable properties of dendrimers, already widely used in the biomedical field, a promising new strategy could be to prepare biodegradable dendrimers functionalized with guanidine residues, with heterocycles having nitrogen atoms with characteristics similar to those of guanidine such as imidazole or with biocompatible molecules containing such residues as arginine and histidine.

## Figures and Tables

**Table 1:** Categories of antimicrobial polymer systems.

Category	Description		Action	Advantages	Drawbacks
biocidal polymer	necessarily cationic	quaternary phosphonium	unspecific electrostatic/disruptive interaction with negatively charged bacteria membranes	no presence of toxic biocide no release of harmful biocides for environmental minor trend to develop resistance	hemolytic toxicity fast clearance from circulation high uptake in the reticuloendothelial system
		guanidinium			
		tertiary sulfonium			
		primary, secondary, tertiary, quaternary ammonium			
	biocidal polymers embodied by the entire macromolecule not requiring biocidal monomers				
polymeric biocide	from polymerization of antimicrobial monomers unnecessary cationic presence of repeated antimicrobial functionalities		same action of the attached biocide moieties	lower systemic toxicity lower hemolytic toxicity lower clearance	less active than free biocide drugs for steric hindrance cause by polymer
biocide-releasing polymer	unnecessary cationic not intrinsic activity of polymer presence of loaded cleavable antimicrobial drugs covalently linked or by physically entrapped		by releasing the entrapped or bond antimicrobial drugs	target release of biocide higher concentration of biocide at the target site excellent efficacy	significant reduction of activity in time toxicity of free biocide

**Table 2:** Examples of Gram-negative bacteria.

Family	Genus	Species
Campylobacteraceae	*Campylobacter*	*Campylobacter coli* *Campylobacter concisus* *Campylobacter jejuni* *Campylobacter rectus*
	*Arcobacter*	*Arcobacter butzleri* *Arcobacter cryaerophilus*
Enterobacteriaceae	*Citrobacter*	*Citrobacter amalonaticus* *Citrobacter braakii* *Citrobacter farmeri* *Citrobacter freundii* *Citrobacter gillenii* *Citrobacter koseri*
	*Enterobacter*	*Enterobacter aerogenes* *Enterobacter agglomerans* *Enterobacter cloacae* *Enterobacter cowanii* *Enterobacter gergoviae*
	*Escherichia*	*Escherichia coli*
	*Klebsiella*	*Klebsiella pneumoniae*
	*Morganella*	*Morganella morganii*
	*Proteus*	*Proteus vulgaris* *Proteus mirabilis*
	*Shigella*	*Shigella dissenteriae*
	*Salmonella*	*Salmonella tiphy*
	*Yersinia*	*Yersinia pestis* (responsible for the plague) *Yersinia pseudotuberculosis* *Yersinia enterocoliti*ca
	*Serratia*	*Serratia marcescens*
	*Aerobacter*	*Aerobacter aerogenes*
	*Enterobacter*	*Enterobacter sakazakii*
Moraxellaceae	*Acinetobacter*	*Acinetobacter baumannii* *Acinetobacter beijerinckii* *Acinetobacter bereziniae* *Acinetobacter boissieri*
	*Moraxella*	*Moraxella catarrhalis (Branhamella catarrhalis)*
Neisseriaceae	*Neisseria*	*Neisseria meningitidis*
	*Hemophilus*	*Hemophilus influenzae*
Pasteurellaceae	*Pasteurella*	*Pasteurella multocida*
Pseudomonadaceae	*Pseudomonas*	*Pseudomonas aeruginosa*
Vibrionaceae	*Vibrio*	*Vibrio cholerae* (responsible for cholera) *Vibrio fischeri* *Stenotrophomonas maltophilia*

**Table 3:** Characteristics of Gram-negative bacteria cell walls.

Possible Constituents of the Bacteria Outer Envelope	Gram-Negative Bacteria	Features	Components	
inner cell cytoplasmic membrane (CM)	present	negatively charged	phospholipid bilayer functional membrane proteinsenzymes	
peptidoglycan layer	present	much thicker that in Gram-positive bacteria	sugars (*N*-acetylglucosamine, *N*-acetylmuramic acid) amino acids (tetrapeptides)	
outer membrane (OM)	present	high density of negative charges	lipopolysaccharide (outer leaflet)	lipid A
				polysaccharide core
				O antigen
			phospholipids membrane proteins	
			lipoproteins (attached to polysaccharide backbone)	single-layer phospholipid
				hydrophilic proteins
			porins	pores for particular molecules
periplasmic space	present	concentrated gel-like substance	periplasm transport proteins sensory proteins peptidoglycan	
surface layer (S-layer)	present	directly attached to OM	proteins glycoproteins	
flagella	possibly present	four supporting rings instead of two	helical protein flagellin with the shape of a 20-nanometer-thick hollow tube	
lipoteichoic acids teichoic acids	absent	molecules that completely cross the wall linked to phospholipids or to peptidoglycan	polyvalent alcohol polymers bonded together through a phosphate group	
Braun’s lipoprotein	possibly present	link between the OM and the peptidoglycan chain by a covalent bond	hydrophilic protein hydrophobic lipid head	

**Table 4:** Comparison between the interaction steps of small molecule antimicrobial agents and cationic antimicrobial polymers (CAPs).

Step	Small Molecule Antimicrobial Agents	CAPs
initial absorption	weak	strong
diffusion past the cell wall	strong	weak
binding into the membrane	weak	strong
disruption and disintegration of the membrane	weak	strong

**Table 5:** Natural and synthetic antimicrobial cationic polymers active on Gram-negative bacteria developed in the last decades.

Structure of Cationic Polymer	Target Bacteria	Antibacterial Activity Expressed as MIC/NBC (µg/mL) Log Reduction*^§^* Antibacterial Efficiency*^#^*	Drawbacks	Advantage	Sectors of Application/Uses
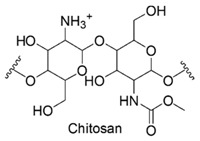 * [42,43]	*E. coli* *X. campestris* *Salmonella enteri*ca *S. tiphymurium* *P. aeruginosa* *Aeromonas hydrophila* *Shigella dysenteriae* *Vibrio cholerae* *V. parahemolyticus* *P. fluorescens* *Enterobacter aerogenes*	20–1000 500 2000–3000 1000–2000 200–1700 1000 > 200 200 150–1000 250–1000 250	difficult control over structure and properties poor reproducibility of results active only at acidic pH non-tuberculocidal non-sporicidal	biocompatible biodegradable available in a large scale low-cost	agriculture sector packaging textile industry biomedicine
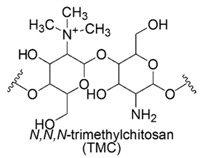 [54,55,56,57,58]	*P. fluorescens* *P. aeruginosa* *E.coli*	> 128 150– > 5000 16–64	difficult control over structure and properties poor reproducibility of results non-tuberculocidal non-sporicidal	biocompatible biodegradable available in a large scale active at every pH value	pharmaceutic biomedical
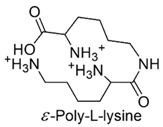 * [49,59,60,61,62,63]	*E. coli K-12* *E. coli F-2* *E. coli B* *E. coli* *P. fluorescens* *P. putida* *P. aeruginosa* *S. marcescens* *S. fonticola* *S. typhimurium*	1–10 2 1 1 100 2–100 3–100 8–100 10 10	difficult control over structure and properties poor reproducibility of results non-tuberculocidal no sporicidal	water soluble biocompatible biodegradable available in a large scale inexpensive low toxicity	food sector antimicrobial food packaging
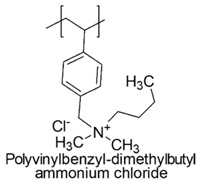 [64,65]	*E. coli* *Aerobacter aerogenes* *P. aeruginosa*	10–33 10–33 66–100	activity reduced by organic material as blood incompatibility with soap non-tuberculocidal no sporicidal	chemical stability non-volatility long-term activity lower toxicity that low MW molecules broad spectrum of activity	disinfection of surfaces disinfection in hospital, nursing homes, public places waters and waste waters treatment macromolecular carrier for antibiotics medical device coatings food packaging industry textiles and fibrous materials industry antimicrobial coatings with wide applications
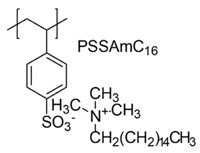 [66]	*P. aeruginosa* *E. coli*	1.5*^§^* 4.0–6.5*^§^*	activity reduced by organic material as blood incompatibility with soap non-tuberculocidal non-sporicidal	chemical stability non-volatility long-term activity lower toxicity that low MW molecules broad spectrum of activity	disinfection of non-critical surfaces antimicrobial coatings for surfaces environments medical device coatings food packaging industry
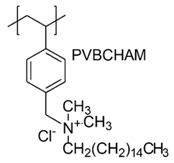 [66]	*P. aeruginosa* *E. coli*	inactive	limited activity activity reduced by organic material as blood incompatibility with soap non-tuberculocidal non-sporicidal	chemical stability non-volatility long-term activity lower toxicity that low MW molecules	disinfection of non-critical surfaces antimicrobial coatings for surfaces environments medical device coatings food packaging industry
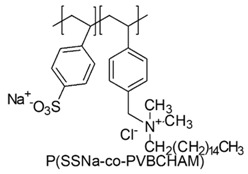 [66]	*P. aeruginosa* *E. coli*	inactive	limited activity poor activity incompatibility with soap	chemical stability non-volatility lower toxicity that low MW molecules	no practical application
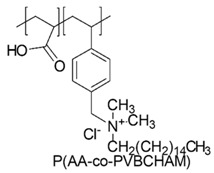 [66]	*P. aeruginosa* *E. coli*	2.5–6.1*^§^* 2.9 *^§^*	activity reduced by organic material as blood incompatibility with soap non-tuberculocidal non-sporicidal	chemical stability non-volatility long-term activity lower toxicity that low MW molecules broad spectrum of activity	disinfection of non-critical surfaces: antimicrobial coatings for surfaces environments medical device coatings food packaging industry
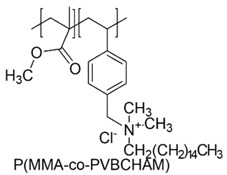 [66]	*P. aeruginosa* *E. coli*	0.8–0.9 *^§^* inactive	limited activity poor activity on Gram-positive incompatibility with soap	chemical stability non-volatility lower toxicity that low MW molecules	no practical application
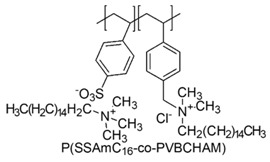 [66]	*P. aeruginosa* *E. coli*	inactive	limited activity activity reduced by organic material as blood incompatibility with soap	chemical stability non-volatility long-term activity lower toxicity that low MW molecules	disinfection of non-critical surfaces antimicrobial coatings for surfaces environments medical device coatings food packaging industry
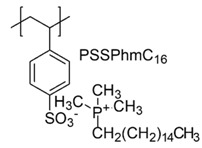 [66]	*P. aeruginosa* *E. coli*	5.7–6.1*^§^* 5.1 *^§^*	activity reduced by organic material as blood incompatibility with soap non-tuberculocidal non-sporicidal	chemical stability non-volatility long-term activity lower toxicity that low MW molecules broad spectrum of activity	disinfection of non-critical surfaces antimicrobial coatings for surfaces environments medical device coatings food packaging industry
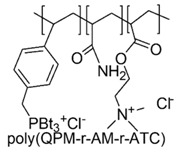 [67]	*E. coli*	3.9–60	activity reduced by organic material as blood non-tuberculocidal non-sporicidal	dual-functional chemical stability non-volatility long-term activity lower toxicity that low MW molecules broad-spectrum	medical device coatings applications in high-hygiene products applications in implants in pulp and papermaking
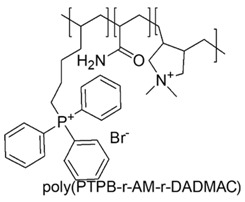 [68]	*E. coli*	75–250 ^1^	activity reduced by organic material as blood limited antimicrobial activity	dual-functional device antiviral chemical stability non-volatility long-term activity lower toxicity that low MW molecules	flocculant and disinfectant for water clarification and sterilization papermaking industry additive in hygiene products
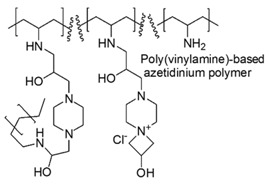 [69,70]	*E. coli*	10–100	activity reduced by organic material as blood incompatibility with soap non-tuberculocidal non-sporicidal	chemical stability non-volatility low hemolytic toxicity	disinfectants medical device coatings food packaging industry antimicrobial coatings with wide applications
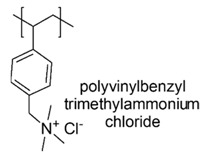 [71]	*E. coli JM109*	> 50 > 50	no activity in liquid-medium assay	antimicrobial activity in an agar-plate assay	disinfectants waters and waste waters treatment macromolecular carrier for antibiotics medical device coatings food packaging industry textiles and fibrous materials industry antimicrobial coatings with wide applications
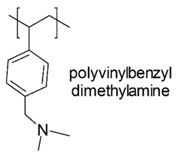 [71]	*E. coli JM109*	25 12.5	highly hemolytic melittin toxin mimic not selective not suitable for clinical uses non-tuberculocidal non-sporicidal	high activity biocidal low-cost	disinfection of non-critical surfaces disinfection of hospital nursing homes public places not suitable for clinical uses
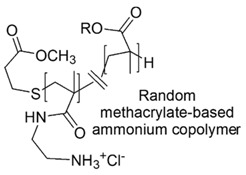 [72]	*E. coli*	8.1–1000 ^2,3^ 7.7–8 ^2,4^	residual cytotoxicity low biocompatibility non-tuberculocidal non-sporicidal	considerable activity chemically stable tunable cytotoxicity low hemolytic toxicity good selectivity possibility of conjugation with other functional groups	food industry hospitals surface coatings that kill bacteria on contact inhibitors of biofouling or biofilm accumulation
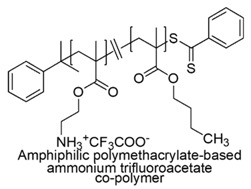 [69,73]	*P. aeruginosa* *E. coli*	4–8 ^5^ 3–4 ^5^	low biocompatibility hemolytic toxicity poor selectivity non-tuberculocidal non-sporicidal	High activity chemically stable	disinfectants not suitable for clinical applications
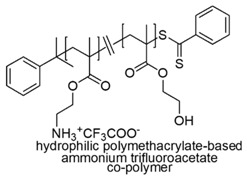 [69,73]	*P. aeruginosa* *E. coli*	16–32 ^5^ 4 ^5^	non-tuberculocidal non-sporicidal	High activity chemically stable High biocompatibility Low Hemolytic toxicity High selectivity	promising antimicrobials with potential for clinical applications
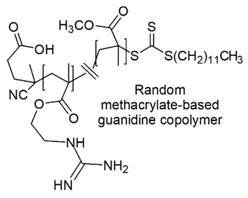 [69,74]	*E. coli*	> 1500	poor activity	Low hemolytic toxicity chemical stability non-volatility long-term activity anti-fungal	promising for producing antimicrobial surface for use in biomedical devices
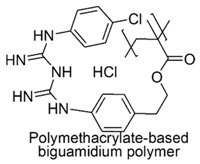 [15,65]	*E. coli*	40	limited spectrum of action non-tuberculocidal non-sporicidal	Non-irritative for skin Non-mutagenic Non-cancerogenic chemical stability non-volatility long-term activity good efficacy	disinfection of non-critical surfaces in hospital, nursing homes, public places
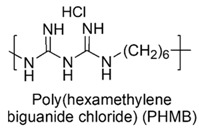 [65,75,76,77]	*E. coli* *E. coli ATCC 8739*	0.29–1.25 ^2^ 1.5–10 ^2,5^ 1.7–4.5 ^2^ 1.8–10 ^2,5^	non-tuberculocidal non-sporicidal	non-irritative for skin non-mutagenic non-cancerogenic chemical stability non-volatility long-term activity high efficacy	*Acanthamoeba keratinitis* treatment beer glass sanitizers general disinfection food industry swimming pools disinfection
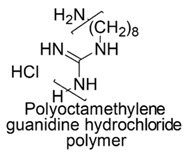 [78]	*E.coli* *Klebsiella* spp. *P. Mirabilis* *Citrobacter* spp. *Citrobacter* ^6^ *Enterobacter* spp. *Enterobacter* spp. ^6^ *Indole-positive proteae* *S. marcescens* *S. marcescens* ^6^ *P. aeruginosa wild type* *P. aeruginosa* ^7^ *Acinetobacter* spp. *Acinetobacter* spp.^8^ *S. maltophilia*	2–8 2–8 4–16 2–8 1–4 2–4 2–4 8–16 4–8 16 4–16 8–16 8–16 8–16 2–16	non-tuberculocidal non-sporicidal	high antibacterial activity broad spectrum of action high activity against drug resistant bacteria bactericidal at low dosage	permanent sterile-surface materials for hospital infection control
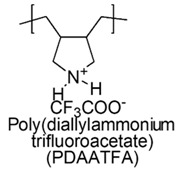 [79,80]	*E.coli ATCC* *P. aeruginosa ATCC* *K. pneumoniae ATCC* *P. mirabilis*	15–125 ^1,5^ 125 ^5^ 15 ^5^ 31^5^	non-tuberculocidal non-sporicidal	chemical stability non-volatility long-term activity lower toxicity that low MW molecules broad spectrum of activity	for use in areas of medicine means to fight infection food industry prevention of bacterial contamination water sanitation
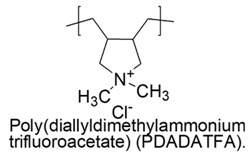 [79,80,81]	*E.coli ATCC* *P. aeruginosa ATCC* *K. pneumoniae ATCC 1* *P. mirabilis*	7–62 ^1,5^ 31 ^5^ 62 ^5^ 31 ^5^	non-tuberculocidal non-sporicidal	chemical stability non-volatility long-term activity lower toxicity that low MW molecules broad spectrum of activity	for use in areas of medicine means to fight infection food industry prevention of bacterial contamination water sanitation
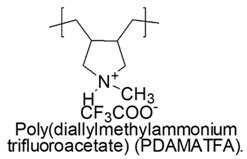 [80,81,82,83,84]	*E. coli ATCC* *P. aeruginosa* *P. aeruginosa MDR* *K. pneumoniae KPC+*	5 ^5^ 1–2 ^5^ 1.5 ^9^ 0.9 ^9^	non-tuberculocidal non-sporicidal	no hemolytic activity high selectivity biocidal activity chemical stability non-volatility long-term activity low susceptibility to resistance broad spectrum of activity	for use in areas of medicine means to fight infection food industry prevention of bacterial contamination water sanitation vegetable oils purification selective removal of free fatty acids from oils and fats
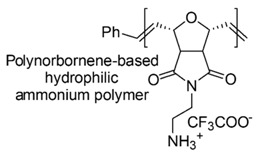 [85]	*E. coli*	400	poor activity	high selectivity low hemolytic toxicity	not signaled
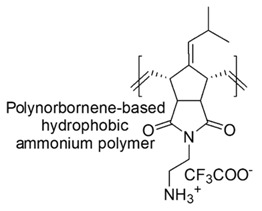 [86]	*E. coli* *S. marcescens*	25 25	low selectivity high hemolytic toxicity non-tuberculocidal non-sporicidal	high ability in inserting in CM high ability in disrupting CM high effectiveness	potential antimicrobial agents with low clinical applicability
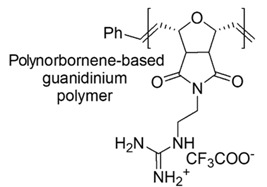 [87]	*E. coli* *S. marcescens*	6 50	non-tuberculocidal non-sporicidal	bactericidal broad spectrum high ability in inserting in CM high ability in disrupting CM high activity low hemolytic toxicity high selectivity	biomedicine disinfection of cardiovascular implants orthopedic implants
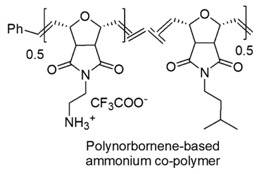 [85]	*E. coli*	50	less active than magainin (AMP) considerable residual hemolytic toxicity Poor selectivity non-tuberculocidal non-sporicidal	tunable activity tunable cytotoxicity tunable selectivity depending on HLB	potential antimicrobial agents
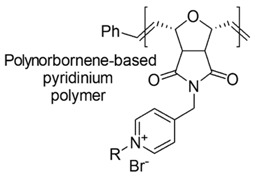 [88]	*E. coli*	4–200	high cytotoxicity for achieving good activity non-tuberculocidal non-sporicidal	tunable activity tunable selectivity	potential antimicrobial agents with low clinical applicability
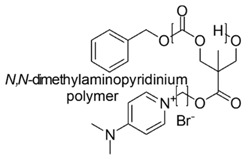 [69,89]	*E. coli* *P. aeruginosa*	31–63 31–125	poor activity against fungi poor activity against *P. aeruginosa* non-tuberculocidal non-sporicidal	broad spectrum good activity on *E. coli* low hemolytic toxicity high selectivity	development of antimicrobial agents for clinical applications
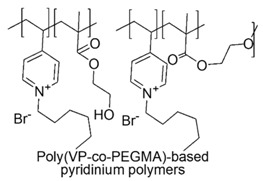 [69,90]	*E. coli*	5–70 ^5,10^	biocompatibility depending on VP content hemolytic toxicity depending on VP content ineffective if highly biocompatible non-tuberculocidal non-sporicidal	tunable biocompatibility tunable hemolytic toxicity acceptable bactericidal activity water-solubility good wettability	permanent bactericidal-surface materials for hospital infection control antimicrobial coatings
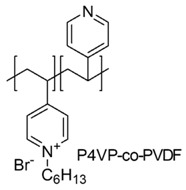 [91]	*E. coli*	8	non-tuberculocidal non-sporicidal	tunable bactericidal activity low susceptibility to arise resistance high stability reusability	sterile-surface materials to kill air- and waterborne pathogens permanent bactericidal-surface materials for controlling hospital infection antimicrobial coatings antimicrobial beads
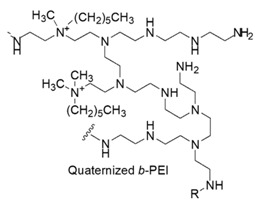 [52,53]	*E. coli* *P. aeruginosa*	74–96 (glass) 70–95 (NPs) 99 (cotton) 98 (wool) 99 (nylon) 96 (polyester) 73–97 (glass) 67–96 (NPs) 98 (cotton) 97 (wool) 98 (nylon) 98 (polyester)	non-tuberculocidal non-sporicidal	significant to total bactericidal activity no toxicity no release of LPS reusable after washing	permanent bactericidal-surface materials for hospital infection control antimicrobial coatings food industry prevention of bacterial contamination water sanitation antibacterial paints and fillers textile industry
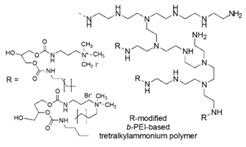 [92]	*E. coli*	60–100	non-tuberculocidal no sporicidal poor activity high hemolytic toxicity poor solubility	chemical stability non-volatility	not suitable for clinical applications poor applicability

*Natural polymers; ^§^ antimicrobial activity is expressed as log reduction of bacteria population; ^#^ activity is expressed as antibacterial efficiency (%); ^1^ depending on PTPB content; ^2^ depending on MW; ^3^ R = methyl; ^4^ R = butyl; ^5^ MBC is given; ^6^ ceftazidime resistant; ^7^ multidrug resistant; ^8^ ciprofloxacin and levofloxacin resistant; ^9^ the minimal concentration for reduction of CFU counting to one; ^10^ depending on VP content.

**Table 6:** Variations in the antimicrobial activity of some CAPs in function of variations in MW and alkyl chains length.

Type of CAPs	MW (Da)	Alkylation	Antimicrobial Activity
oligomeric guanidinium	↓MW		↓activity [130]
quaternized *N*-hexyl-PVP, immobilized on a surface	160,000		full bactericidal effect [131]
quaternized *N*-hexyl, *N*-methyl-PEI immobilized on a surface	25,000		full bactericidal effect [131]
quaternized poly(2-(dimethylamino)ethyl methacrylate) (PDMEMA)	Mn >10,000		100% killing efficiency [110]
secondary and tertiary polydiallylamines (PDAAs)	↑MW		↓MIC100 [79]
poly alkyldimethyl(vinylbenzyl) ammonium chloride		alkyl C_12_ chain	↑activity [64]
cationic quaternary copolymers (vinylamine, aminoalkyl methacrylates, and *N*-vinyl pyrrolidone) with pendent quaternary ammonium groups		any length	not influenced by the length of the alkyl substituents at the nitrogen [132]
alkylated quaternized PVP polymers		alkyl > C_6_ chain	↓activity [133]
quaternized poly (4-vinyl pyridine) (P4VP)-poly (vinylidene fluoride) (PVDF) co-polymer		C_4_>chain< C_10_	↑activity [134]
quaternized alkyl pyridinium polyoxanorbornene		Chain < C_4_	minimal activity [88]
quaternized alkyl pyridinium polyoxanorbornene		Chain > C_6_	↑activity [88]

**Table 7:** Correlations between polymers structural properties, antimicrobial activity and toxicity.

Type of CAP	CAP Structural Properties	Hemolysis (H) Cytotoxicity (C) Biocompatibility (B)	Antimicrobial Activity
quaternized pyridinium–methacrylate copolymers	charge spatially separates by alkyl tails	↑H ↑C	↑ [134]
hydrophilic vinylpyridine-based co-polymer quaternized with PVP links	use of strongly hydrophilic comonomers (HEMA or PEGMA)	↓H,C	↑ [90]
quaternized PVP	use of hydrophilic comonomers	↓H ↑B	↑ [125]
*N*-hexyl, methyl-PEI	larger size	no appreciable	↑ [135]
polystyrene-based ammonium polymers	protonated tertiary amine groups	not available	↑ [71]
polystyrene-based ammonium polymers	quaternized ammonium groups	not available	↓ [71]^ 1^
random amphiphilic methacrylamide-based ammonium copolymers	protonated primary amine groups hydrophobic alkyl groups in the side chains	minimal	↑ [72]
random amphiphilic methacrylamide-based ammonium copolymers	protonated tertiary amine groups hydrophobic alkyl groups in the side chains	minimal	↑ [72]
random amphiphilic methacrylamide-based ammonium copolymers	quaternized ammonium groups hydrophobic alkyl groups in the side chains	considerable	↓ [72]
Water-soluble cationic PDAAs containing pyrrolidine links	protonated secondary or tertiary amine groups	not available	↑ [136]
quaternized ammonium groups	quaternized ammonium groups	not available	↓ [79]
poly(diallylammonium trifluoroacetate) (PDAATFA)	protonated secondary amine groups	not available	↑ [79]
poly(diallylammonium trifluoroacetate) (PQAS TFA)	quaternary ammonium groups	not available	↓ [79]^ 1^
quaternized alkyl pyridinium polyoxanorbornene	chain < C_4_	low	↓ [88]
quaternized alkyl pyridinium polyoxanorbornene	chain > C_6_	considerable	↑ [88]

^1^ compared to the not quaternized polymers reported in the previous row.

## Abbreviations

2-(acryloyloxy)ethyltrimethylammonium chloride, ATC; 2-2’-azobisisobutirronitrile, AIBN; 2-hydroxyethyl methacrylate, HEMA; 4-(2,5-dioxo-pyrrolidin-1-yloxycarbonyl)-benzyl)-triphenyl-phosphonium bromides, NHS–QPS; 4-penten-1-yl-triphenylphosphonium bromide, PTBT; 4-vinylpyridine, 4VP; acrylamide, AM; acrylic acid, AA; antibiotic-resistant bacteria, ARB; antibiotic-susceptible bacteria, ASB; benzyl bromide, BzBr; benzyl-N-quaternary chitosan ammonium salt, BNQAS–CS; branched polyethyleneimine, *b*-PEI; cationic antimicrobial peptides, CAMPs; cationic antimicrobial polymers, CAPs; colony-forming unit, CFU; cytoplasmic membrane, CM; diallyl dimethyl ammonium chloride, DADMAC; dimethylamine pyridine, DMAP; divinylbenzene, DVB; dodecyl quaternary chitosan ammonium salt, C_12_QAS–CS; effective concentration, EC; extensively drug resistant, XDR; facially amphiphilic, FA; free radical polymerization, FRP; halohydrocarbons, RX; hemolytic cytotoxicity, HC; hexadecyl quaternary chitosan ammonium salt, C_16_QAS–CS; hexyl bromide, HB; high-density polyethylene, HDPE; kilodalton, kDa; lipopolysaccharide, LPS; low-density poly ethylene, LDPE; minimum bactericidal concentration, MBC; minimum inhibitory concentration, MIC; molecular weight, MW; multidrug-resistant, MDR; *N,N,N*-trimethylchitosan, TMC; nicotinamide adenine dinucleotide oxidoreductases type 2, NDH-2; *N*-quaternary phosphonium chitosan derivatives, New Delhi metallo-beta-lactamase 1, NDM-1; *N*-QPCSxy; octadecyl quaternary chitosan ammonium salt, C_18_QAS–CS; *O*-quaternary chitosan ammonium salt, QAS–CS; outer membrane, OM; poly(acrylamide-co-VBCHAM), P(AA-co-VBCHAM); poly(cetyltrimethylammonium-4-styrene) sodium sulfonate-co-VBCHAM, P(SSNa-co-VBCHAM); poly(cetyltrimethylammonium-4-styrene)sulfonate, PSSAmC_16_; poly(cetyltrimethylammonium-4-styrene)sulfonate-co-VBCHAM), P(SSAmC_16_-co-VBCHAM); poly(cetyltrimethylphosphonium-4-styrene)sulfonate, PSSPhC_16_; poly(diallylamines), PDAAs; poly(diallylammonium) trifluoroacetate, PDAATFA; poly(diallyldimethyl) ammonium chloride, PDDA; poly(diallyldimethylammonium trifluoroacetate, PDADMATFA; poly(diallylmethylammonium trifluoroacetate, PDAMATFA; poly(ethylene glycol) methyl ether methacrylate, PEGMA; poly(ethylene terephthalate), PET; poly(hexamethylene) biguanidine chloride, PHMB; poly(VBCHAM)-co-poly(methymetacrylate-co-VBCHAM, P(MMA-co-VBCHAM);poly(vinylidene)fluoride, PVDF; poly(vinyl-*N*-hexyl pyridinium bromide, hexyl-PVP; poly-4-vinyl pyridine, P4VP; polyethylene glycol, PEG; polyguanidinium oxanorbornene, PGON; polyhexamethylene guanidine hydrochloride, PHMG; polymeric biguanidine salts, PBGSs; polymeric guanidine, PGSs; polymeric quaternary ammonium salts, PQASs; polymeric quaternary phosphonium salts, PQPSs; polynorboranes, PNBs; polyoctamethylene guanidine hydrochloride, POMG; polypropylene, PP; polysulfone, PSF; quaternary phosphonium macromonomer, QPM; random, r; red blood cells, RBC; reversible addition-fragmentation chain transfer polymerization, RAFT; reversible deactivation radical polymerization, RDRP; ring-opening metathesis polymerization, ROMP; ring-opening polymerization, ROP; sodium dodecyl sulfate, SDS; styrene, St; synthetic antimicrobial peptide, SAMP; *tert*-butyloxycarbonyl, BOC; tetradecyl quaternary chitosan ammonium salt, C_14_QAS–CS; transmission electron microscopy, TEM; tributyl(4-vinylbenzyl)phosphonium, QPM; trimethylamine, TMA; triphenyl butyl phosphonium, PTPB; vinylbenzyl dimethylhexadecylammonium chloride, VBCHAM; World Health Organization, WHO; *ε*-poly-*L*-lysine, *ε*-PL.
